# Two metrics for quantifying systematic errors in diffraction experiments: systematic errors in the variance of the observed intensities and agreement factor gap

**DOI:** 10.1107/S1600576725004376

**Published:** 2025-06-20

**Authors:** Julian Henn

**Affiliations:** a DataQ Intelligence, Fichtelgebirgsstrasse 66, 95448 Bayreuth, Germany; The University of Western Australia, Australia

**Keywords:** systematic errors, metrics, twinning, disorder

## Abstract

The increase in the weighted agreement factor due to systematic errors in single-crystal X-ray and neutron diffraction experiments can be quantified precisely, provided the estimated standard uncertainties of the observed intensities, s.u.(*I*_obs_), are sufficiently accurate. The increase in the weighted agreement factor quantifies the ‘costs’ of the systematic errors. This is achieved by comparison with the lowest possible weighted agreement factor for the specific data set. Application to 314 published data sets from inorganic, metal–organic and organic compounds shows that systematic errors increase the weighted agreement factor by a surprisingly large factor of *g* = 3.31 (or more) in 50% of the small-molecule data sets from the sample. Examples of twinning, disorder, neglect of bonding densities and low-energy contamination are taken from the literature and examined with respect to the increase in the weighted agreement factor, which is typically less than three. The large value *g* = 3.31 for the supposedly simple case of rather small molecules, as opposed to macromolecules, is interpreted as a warning sign that there are not only the expected remaining systematic errors, like not-modelled disorder, unrecognized twinning or neglect of bonding electrons or similar errors, but additionally a common systematic error of insufficiently accurate s.u.(*I*_obs_). Inadequate s.u.(*I*_obs_) may not just compromise the model parameters and model parameter errors; they are also a threat to the whole data quality evaluation procedure that relies crucially on adequate s.u.(*I*_obs_).

## Abstract 2

The weighted agreement factor in small-molecule crystallography is, for half of a sample (*N* = 314) of published data sets, so large that it cannot be explained by errors like unrecognized disorder or twinning. Instead, the standard uncertainties are most likely flawed in these cases.

## 1. Introduction

The assessment of measurement methods and evaluation procedures is an important part of the scientific method. To this end it is also necessary to quantify the degree of systematic error in any given crystallographic data set. Appropriate metrics specifically for this purpose are needed. A variety of metrics for the detection, quantification or visualization of systematic errors or for the quality of results in single-crystal diffraction experiments are already available, like the normal probability plot npp (Abrahams & Keve, 1971 ▸), the Diederichs plot (Diederichs, 2010 ▸), the redundancy-independent merging factor *R*_r.i.m._ and precision-indicating merging factor *R*_p.i.m._ (Weiss, 2001 ▸), checkCIF procedures (Spek, 2003 ▸; Spek, 2009 ▸; Spek, 2018 ▸; Spek, 2020 ▸) based on the CIF standard (Hall *et al.*, 1991 ▸), and alert systems from software like *PLATON* (Spek, 2020 ▸). Each of these metrics and procedures comes with individual limitations and advantages, but it is out of the scope of this article to discuss all of these in great detail. Therefore, only a small selection will be briefly discussed in the following paragraph.

The npp visualizes deviations in the distribution of weighted residuals from the ideal case. The question of the origin of these deviations is not answered by the npp; possible main causes are structure model deficiencies or inadequate weights. The Diederichs plot may be used to quantify the systematic instrument error by evaluation of the maximum significance max[*I*_obs_/σ(*I*_obs_)]. Its reciprocal value is related to the merging *R* factor. Diederichs concludes that ‘the accuracy of data at low resolution is usually limited by the experimental setup rather than by the crystal’ (Diederichs, 2010 ▸). The disadvantage of the merging *R* factor *R*_merge_ (Stout & Jensen, 1989 ▸; Blundell & Johnson, 1976 ▸; Drenth, 2007 ▸) to show lower values for less redundant data^1^ was overcome by the introduction of the merging agreement factor *R*_r.i.m._ (Weiss & Hilgenfeld, 1997 ▸; Weiss *et al.*, 1998 ▸), which is also called *R*_meas_ (Diederichs & Karplus, 1997 ▸). More merging *R* factors are found in the literature. A common disadvantage of *R*_merge_, *R*_r.i.m._ and *R*_p.i.m._ is that they all lead to lower values, thus indicating higher quality, when the observed intensities are overestimated, which follows from the respective definitions. For a correct interpretation of these merging *R* factors it is therefore important to exclude overestimation of observed intensities. Note that even a slight overestimation of *I*_obs_ on average may influence the merging *R* factors considerably, as the abundant weak data show the largest merging *R* factor and these are most strongly affected by a slight overestimation of *I*_obs_.

None of these briefly discussed metrics gives direct clues to the origin of errors. Indeed, they need not from the subjective perspective of this author, as it is deemed to be a valid approach to separate the quantification and visualization of systematic errors, which may already be a difficult task, from finding the sources of systematic errors, which is often a much harder task. Improving the ability to characterize, quantify and discriminate between the appearance of systematic errors in the data is, in the personal view of the author, an important objective in itself for long-term progress in crystallography, very similar to good diagnostics being important for questions of health, despite the fact that a diagnosis in itself does not cure any disease. It helps, however, to discriminate between similar illnesses, which may need completely different treatments. This ensures that the cure is not more harmful than the disease.

In macromolecular crystallography, the ratio of the ‘working conventional agreement factor’ and the ‘free *R* value’, the conventional agreement factor of a test set from the observed intensities excluded from the refinement, is taken for cross validation (Bruenger, 1992 ▸). The concept of the free *R* value has also received some criticisms and modifications. Holton *et al.* (2014 ▸) define an ‘*R*-factor gap in macromolecular crystallography’ by comparison of the merging *R* factor and the conventional agreement factor *R*. They suggest that ‘the reason for high *R* factors in macromolecular crystallography is neither experimental error nor phase bias, but rather an underlying inadequacy in the models used to explain our observations.’

As the weighted agreement factor is a very common and popular metric, in particular for small-molecule crystallography, it is rather surprising that there is not yet a metric in use that quantifies the increase in the weighted agreement factor in small-molecule crystallography due to the presence of systematic errors or due to systematic errors in the variance of the observed intensities, two connected metrics suggested in this work. The concept of a predicted weighted agreement factor was suggested earlier (Henn & Schönleber, 2013 ▸; Henn & Meindl, 2014*a* ▸; Henn & Meindl, 2014*b* ▸; Henn, 2018 ▸; Henn, 2014 ▸) and the present article continues this former work by defining how much lower the weighted agreement factor would be in the absence of systematic errors compared with the weighted agreement factor from a model refinement. This metric is easy to grasp, and its development and application serve to stimulate a discussion about systematic errors in small-molecule crystallography. It will be seen later that this value is surprisingly high, which reveals that there are some common fundamental errors not only in macromolecular crystallography but also in small-molecule crystallography.

According to the definition of the International Union of Crystallography (IUCr), systematic errors are the ‘contribution of the deficiencies of the model to the difference between an estimate and the true value of a quantity’.

In single-crystal diffraction, the word ‘model’ usually refers to the structure model, the parameters of which are refined against experimental intensities *I*_obs_ and deliver the model-derived intensities *I*_calc_. However, the observed intensities and the corresponding standard uncertainties are also part of the model according to the definition of the IUCr, as they are constructed from the raw data using assumptions and models regarding the scattering theory, detector properties, background intensities, polarization *etc*.

As a consequence of this definition, and maybe in contrast to intuition, the phrase ‘systematic errors’ leaves the origin of the error open. It may be in the ‘experimental data’, like in the observed intensities *I*_obs_ and their corresponding standard uncertainties s.u.(*I*_obs_), or in the structure model, *i.e.* in *I*_calc_, or in both or *e.g.* in an oversimplified scattering theory. In this case a correct structure model would still lead to systematic differences with correct *I*_obs_. In this work, the definition of the IUCr is adhered to.

Systematic errors are found by comparing observed and structure-model-derived entities like *I*_obs_ and *I*_calc_. When systematic differences are found this clearly indicates the presence of systematic errors, but it does not necessarily reveal anything about the origin of this error.

The word ‘data’ refers in this work mainly to the set of *h*, *k*, *l*, *I*_obs_, s.u.(*I*_obs_) and *I*_calc_,^2^ the information available after model refinement that includes the calculated intensities. For a refinement with the *SHELXL* software (Sheldrick, 2015 ▸) the corresponding data are found in the files ending with ‘.fcf’. By no means is the word ‘data’ limited to *I*_obs_ and s.u.(*I*_obs_).

## 2. Metrics for the quantification of systematic errors

In a data set without any systematic error, a sufficiently sophisticated crystal structure model is refined against the set of observed intensities *I*_obs_ that are known with uncertainty s.u.(*I*_obs_). As a result, a set of corresponding calculated intensities *I*_calc_ are obtained in return. The difference between observed and calculated intensity is divided by the standard uncertainty s.u.(*I*_obs_) in order to obtain the weighted residual ζ = 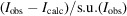. The standard uncertainties are found in the input reflection file. The weighted residual is, in the absence of systematic errors, a random variable. The employment of s.u.(*I*_obs_) in ζ is referred to as ‘statistical weights’.

Many published data sets, however, have weights from a more extended weighting scheme. As an example, equation (1) gives a weighting scheme as implemented in *SHELXL*:
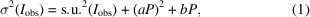
with weighting scheme parameters *a* and *b*, and *P* = 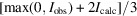. The use of *P* instead of *I*_obs_ was suggested by Wilson (1976 ▸) to reduce statistical bias. When the s.u.(*I*_obs_) are severely underestimated, however, bias is *increased* by this choice (Henn, 2025 ▸). Equation (1) is already a simplified version of the weighting scheme; there are more parameters available in the full form but these are not needed for the present discussion. Sheldrick (2015 ▸) referred to equation (1) as inverse weight, 1/*w* = σ^2^(*I*_obs_), whereas here it is deliberately written as the variance of the observed intensity, σ^2^(*I*_obs_). After all, according to the *SHELXL* manual pages the weighting scheme serves exactly this purpose, ‘so that the variance shows no marked systematic trends with the magnitude of 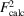 or of resolution’ (https://shelx.uni-goettingen.de/shelxl_html.php#WGHT).

The purpose of the weighting scheme and the requirements for appropriate application of a weighting scheme are of such great importance that we take a moment to elaborate on this topic here. There are two fundamentally distinct cases for the variance of the residuals not being flat:

(i) The variance of the observed intensities s.u.^2^(*I*_obs_) is underestimated for a part of the data. In this case, the variance of the residuals changes with resolution or intensity, even when the structure model is entirely correct and no other error is present. The application of a weighting scheme serves to restore the correct variances. These could and should be used to improve data integration such that adequate variances result in the first place and application of a weighting scheme is not necessary (provided there are no other errors).

(ii) There are other systematic errors present. Two important cases are distinguished: (*a*) A few individual outliers distort the model parameters such that it seems to be appropriate to weight these down in order to prevent distortion of the model parameter values by these outliers. The application of a weighting scheme is frequently discussed just in this context. (*b*) There is a *systematic* – but not necessarily large – deviation *I*_obs_ > *I*_calc_ or *I*_obs_ < *I*_calc_ for a fraction of the data, such that certain bin mean values 〈*I*_obs_〉/〈*I*_calc_〉 ≠ 1 for sufficiently large chosen bins deviate distinctly from one. A fraction of the calculated reflections, such as the weakest 10%, are systematically larger or weaker than the corresponding observed intensities in this case. This constitutes a systematic error already in itself, as a fraction of 10% is for most data sets too large to occur by accident. However, the weighting scheme will only be invoked in *SHELXL* if *additionally* the differences between observed and calculated intensities are frequently much larger than the respective s.u.(*I*_obs_). Application of the weighting scheme effectively disguises the systematic error in this case, as it just makes the variance of the observed intensities so large as to accommodate the formerly significant (and still systematic) differences between observed and calculated intensities. The variance is finally flat and nothing points to the important systematic error.

To make this a bit more concrete, the reader may think for example about not-modelled non-merohedral twinning, which frequently leads to weak intensities being too large and thus to a ratio *K* = 〈*I*_obs_〉/〈*I*_calc_〉 ≫ 1 specifically for the weakest *I*_calc_ (Müller, 2006 ▸). *K* is given in the *SHELXL* output list file. A large value of *K* for low-intensity reflections is typically accompanied by a large weighting scheme parameter value *b* that becomes smaller or vanishes after modelling of twinning. A non-vanishing value *b* > 0, however, indicates the presence of one or more other systematic errors. To make this discussion even more concrete we consider the first example of non-merohedral twinning discussed in ch. 7.8.5 of Müller (2006 ▸) (methylene diphosphonic acid, CH_6_O_6_P_2_). Here, the values in the initial stage of the refinement, where twinning was not yet modelled, were *K* = 11.456 and *b* = 27.6049 (nonm1-02) and in the final stage *K* = 0.365 and *b* = 0.7180 (nonm1-07). Modelling of twinning has thus led to a large reduction in the weighting scheme parameter *b*. We will see later in Table 1 that modelling of twinning decreased the weighted agreement factor in this case by an impressive factor of 2.94. However, because *a*, *b* ≠ 0 in the final stage of the refinement, the agreement factor could be reduced still further by *another* factor of 2.92. In other words, another systematic error remains, which is, in terms of the ratios of agreement factors, of similar magnitude to the twinning. The absolute values are less dramatic but still impressive: modelling of twinning reduces the weighted agreement factor from 31.03 to 10.56% and for *a* = *b* = 0 it is 2.92%. So, *wR*(*F*^2^) = 2.92% is the potential of the data provided there are no systematic errors, but only *wR*(*F*^2^) = 10.56% is realized in this example, which is still much less than the initial *wR*(*F*^2^) = 31.03%. Twinning was here just taken as an example; one could equally well choose other examples like not-modelled disorder.

Whenever the weighting scheme parameters increase the variance of the observed intensities, that is, whenever the weighting scheme parameters *a* and/or *b* are not identical to zero, the induced increase in the variance of the observed intensities should be monitored, quantified and set into proportion. Section 2.2 will provide a metric for that purpose. Increasing the variance of the observed intensities will also reduce the weighted agreement factor and lead in this way to an agreement factor gap. This will be discussed in Section 2.3.

Any given weighting scheme – not only the *SHELXL* type – can be decomposed into the contribution s.u.^2^(*I*_obs_) from the reflection input file and additional contributions. Therefore, the new metric (‘systematic error in the variance of the observed intensity’) as developed in Section 2.2 is not tied to a *SHELXL*-like weighting scheme, which is just used as a widespread and popular example.

A need to apply weights different from statistical weights already confirms the existence of systematic errors. However, the cause remains unclear. A fundamental discrimination between different types of causes was made in the discussion above: (i) inadequate standard uncertainties s.u.(*I*_obs_) as given in the input reflection file, (ii)(*a*) individual outliers and (ii)(*b*) systematic differences between observed and calculated intensities.

This distinction is not currently made despite being important and sensible, as insufficiently accurate s.u.(*I*_obs_) cannot always be adequately corrected for. Inadequate correction of inaccurate standard uncertainties can represent the above-mentioned case of a cure worse than the disease and is described in the literature for the case of uniformly underestimated s.u.(*I*_obs_) (Henn, 2025 ▸).

### 2.1. Another remark on the *SHELXL* weighting scheme

For comparison with other types of weighting schemes like Chebychev polynomials [see, as an example, Carruthers & Watkin (1979 ▸)] and for another reason that will be discussed shortly, it is important not just to compare the numerical values of weighting scheme parameters but also to study the overall effect of the weighting scheme on the variance of the observed intensities. The first statement is self-evident, as other weighting schemes may have a very different parameterization and therefore they may not have a parameter equivalent to a weighting scheme parameter *a* or *b* (or any other from the *SHELXL* type of weighting scheme), so they cannot be compared directly. Even if they have a similar parameter, different weighting schemes may involve a different number of parameters, which is again an obstacle to comparison.

The other reason is more subtle and is tied to a *SHELXL*-like weighting scheme: all individual contributions to σ^2^(*I*_obs_) in equation (1) are quadratic except for the term connected to *b*. This has a strange and not very obvious consequence: structures with lower scattering mass *F*_000_ will tend to have larger values of *b*. As a result the weighting scheme parameter value *b* is dependent not only on systematic errors but also on the total scattering mass as expressed by *F*_000_. In the following, a *Gedankenexperiment* is discussed in order to make this unexpected dependence visible and to understand it. For this *Gedankenexperiment* one needs to keep in mind that the scaling of the data is arbitrary, since it follows a convention rather than any natural law. One special scale is when the intensity of the individual reflections is given in photons like with scintillation detectors. Every scale needs to be in proportion to the count of photons, but in this special choice the factor of proportionality is equal to one.

Dimensionless physical properties like the mean significance of the data *must not* depend on scaling as they express a physical reality that is not dependent on the units used for the measurement. If the length of a wall is twice its height, it will be so regardless of whether the distances are measured in ångströms, inches, feet, metres or lightyears.

Now assume that the refinement of a model against observed data *I*_obs,1_, s.u.(*I*_obs,1_) results in *a*_1_ = 0, *b* = *b*_1_, such that 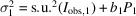. The results of this discussion do not depend on *a*_1_ = 0, but it simplifies the discussion. In *SHELXL*, the scaling of the observed intensities is tied to *F*_000_. The resulting mean significance of the data is given by 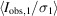. Now, after the refinement is finished we want to scale to 0.5*F*_000_ instead of *F*_000_ for whatever reason (for instance, a friend developed their own refinement software and just chose this scale out of curiosity). The intensities and standard uncertainties expressed in the new scale get an index 2 and they just double, since the scale factor is applied by division: 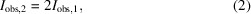

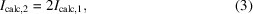

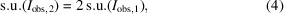

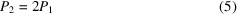
Equation (5) follows from the definition *P* = *f* max(0, *I*_obs_) + (1 − *f*)*I*_calc_ and equations (2) and (3). It holds for any value of *f* ∈ [0, 1]. The dimensionless physical properties *must not* change as no physical change has been applied, only a change of the units, so 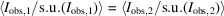 must – and evidently does – hold, as can be seen from equations (3) and (4). However, it is a requirement that 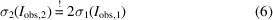
in order to obey 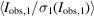
 
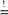
 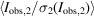, where the exclamation mark above the equals sign symbolizes that equality between the term on the left-hand side and the term on the right-hand side is demanded. Using the definition in equation (1) and equations (2)–(5) in equation (6), one arrives after a very short calculation at 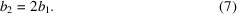


For the rescaled data we need to chose a weighting scheme parameter *b* twice as large as the original one in order to arrive at the same mean significance of the data. This is in contrast to the weighting scheme parameter *a*, which does *not* change under rescaling. If the weighting scheme parameters *a* and *b* described solely the data quality, they would *both* be unchanged under a change of scale, as the data quality is not affected by a change of scale. However, the weighting scheme parameter *b* obviously depends on the applied scale.

The consequences of this argument are as follows. The preceding paragraphs show that the weighting scheme parameter *b* is intimately connected to the scale, whereas the weighting scheme parameter *a* is not. As a consequence, the weighting scheme parameter *b* is comparable only for refinements with the same or a very similar scale factor *F*_000_, *i.e.* for different model refinements with constant *F*_000_ against the same data, but not for entirely different structures. For some hypothetical structures labelled 1–3 taken from a crystallographic databank and having the same weighting scheme parameter *a*_1_ = *a*_2_ = *a*_3_, structure 1 with *F*_000,1_ = 1000, *b*_1_ = 1 is comparable with structure 2 with *F*_000,2_ = 500, *b*_2_ = 2 and with structure 3 with *F*_000,3_ = 250, *b*_3_ = 4. A smaller numerical value of *b* does not automatically imply a better least-squares fit; it depends additionally on *F*_000_. A basic requirement for a metric describing data quality is independence from the scale, which applies to weighting scheme parameter *a* but not to *b*. As a consequence, instead of comparing the numerical values of the weighting scheme parameters directly, it is more objective to compare how the weighting scheme parameters affect the variance of the observed intensities, as will be described in the next section. This approach has the advantage of being independent of the scale and additionally facilitates comparison between different weighting scheme types.

### 2.2. Systematic error in the variance of the observed intensity

The variance of the observed intensity σ^2^(*I*_obs_) can be broken down into a statistical part and a systematic part, where s.u.^2^(*I*_obs_) is the variance due to stochastic error and *X*^2^ is that due to systematic error: 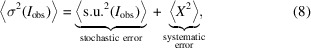

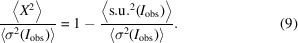
The angle brackets in equations (8) and (9) indicate averaging over the data set. Equation (9) defines the fraction of sys­tematic error in the variance of the observed intensity. It is a positive number ranging between zero and one. For statistical weights with *a* = *b* = 0, it follows that 〈*X*^2^(*I*_obs_)〉/〈σ^2^(*I*_obs_)〉 = 0, indicating that 100% of the variance in the observed intensity is due to stochastic fluctuations. For values of the weighting scheme parameters different from zero, *a* ≠ 0 and/or *b* ≠ 0, the stochastic part is reduced and a systematic error enters, such that both numbers always add up to 100%. This parameteri­zation is taken as a convenient measure to quantify the degree to which systematic errors affect or even dominate the average variance of the observed intensity 〈σ^2^(*I*_obs_)〉 in a given data set. [It may very well be that equations similar to equation (9) were discussed previously in the literature, but not to the knowledge of the author.] The significance of this number lies in the fact that (i) it enables us to define intuitive threshold values for high-quality data sets based on a convention and (ii) this threshold value is based on the effect of the weighting scheme on 〈σ^2^(*I*_obs_)〉 rather than being based on weighting scheme parameter values. In this way different weighting schemes can easily be compared with each other.

**Figure 1:**
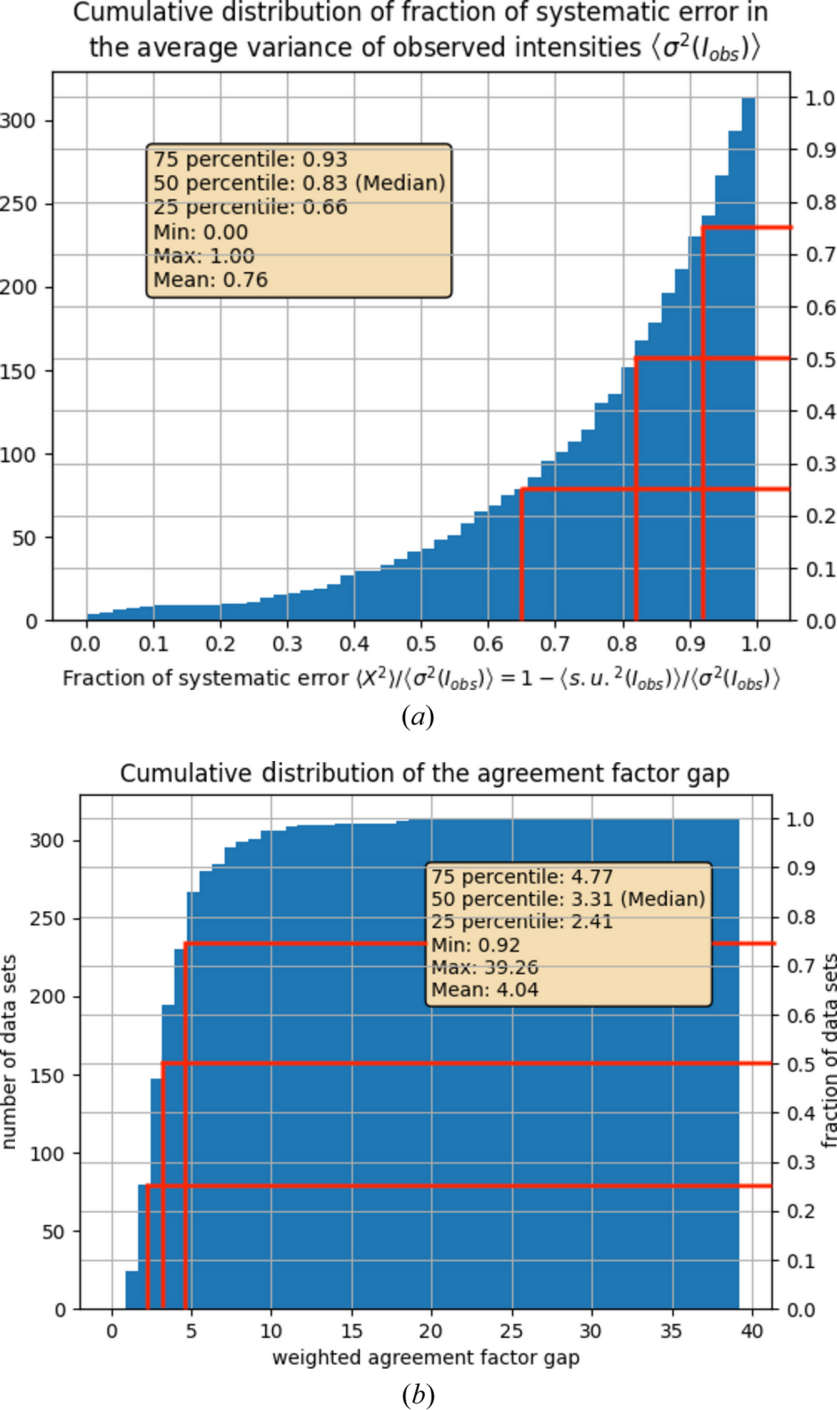
(*a*) Cumulative distribution of the fraction of systematic error 〈*X*^2^〉/〈σ^2^(*I*_obs_)〉 in the variance of the observed intensities for 314 data sets. Only 25% of the data sets have a fraction of systematic error in the variance of the observed intensities of 66% or less. (*b*) Cumulative distribution of the agreement factor gap as defined in equation (11). For 50% of all data sets the weighted agreement factor is increased by a factor of 3.31 or more as a consequence of systematic errors. For more information see the text.

As an example for (i), one might define data sets with 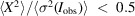, *i.e.* data sets with less than 50% contamination of systematic errors in the variance of the observed intensities, to be of high quality. If this definition appears to be quite generous to the reader, they will probably be surprised to learn that less than 20% of all data sets in our sample of *N* = 314 small-molecule data sets conform to this requirement [see Fig. 1(*a*) in Section 3]. This result can be interpreted in very different ways. (i) Either the s.u.(*I*_obs_) are so small that even small errors like slight disorder or not-modelled bonding density are detected and lead to a large increase in the weighted agreement factor, or (ii) the s.u.(*I*_obs_) are just *too* small and do not describe the variance in the observed data adequately. Interpretation (i) assumes a very high precision of the experimental data, while interpretation (ii) assumes that the high precision of the data is exaggerated and only of a formal nature and is not physically realized. In case (i), the task for enabling further progress in data quality would be to identify and remove those slight errors, and in case (ii) the task would be to learn how to obtain correct s.u.(*I*_obs_) in the first place. The often tacitly assumed notion that the weighting scheme enables corrections in a meaningful way may not necessarily hold in any individual case, and it certainly does not hold in the simple case that all s.u.(*I*_obs_) are underestimated by a common factor (Henn, 2025 ▸).

### 2.3. Agreement factor gap

The weighted agreement factor is designed to measure the overall difference between the structure-model-derived entity *I*_calc_ as obtained after a least-squares refinement of a crystal structure model against observed intensities and those observed reflection intensities *I*_obs_. Random deviations are characterized by being of the order of magnitude of the individual s.u.(*I*_obs_) and typically even within the limits of only one or a few standard deviations, which is a reasonable measure if the s.u.(*I*_obs_) describe the actual fluctuations in *I*_obs_. Systematic errors may lead to larger deviations, which leads to invoking a weighting scheme in order to ensure the model parameter values are not overly aligned with the strongest outliers. This increases the variance of the observed intensities which in turn increases the weighted agreement factor.

The extent to which the agreement factor is increased in total due to systematic errors can be quantified by dividing the post-refinement weighted agreement factor *wR*(*F*^2^) by a reference value 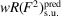. The reference value is the weighted agreement factor in the absence of systematic errors, *i.e.* the adequacy of the structure model *and* of s.u.(*I*_obs_) and *I*_obs_ is assumed: 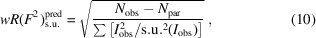
with the number of included reflections in the refinement *N*_obs_ and the number of model parameters *N*_par_.

The ratio 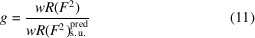
with the post-refinement weighted agreement factor 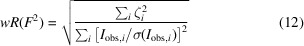
gives the factor by which the agreement factor is increased due to systematic errors. A difference from *g* = 1 implies a gap. The summation index *i* runs over all Miller triples involved in the refinement and 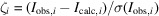 is the weighted residual *i*. The entity defined in equation (11) may be termed the ‘weighted agreement factor gap’ in small-molecule crystallography and is therefore abbreviated here as *g* for ‘gap’. The agreement factor gap is also reported in the checkCIF procedure and may lead to a PLAT969 type *PLATON* message in the CIF report. Our next stage of application to published data sets will show that *g* = 3.31 or larger for half of all sets in our sample of *N* = 314 published data sets.

## 3. Application to published data sets

All data sets published through a peer-review process in the open-access journal *IUCrData* between 2020 and 2022 were examined. This comprises metal–organic, organic and in­organic compounds. Most data sets were collected with Mo or Cu radiation. Data sets that needed editing or were incomplete were excluded. Some publications were just corrigenda without experimental data (Fang *et al.*, 2020 ▸; MacNeil *et al.*, 2020 ▸; Naveen *et al.*, 2021 ▸). In one publication an unusual format of the embedded diffraction data was used (Patel *et al.*, 2020 ▸), in one publication Chebychev polynomials were used (Peña Hueso *et al.*, 2022 ▸) and in some data sets the calculated intensities were not given (de Freitas *et al.*, 2020 ▸; Zhang *et al.*, 2020 ▸; Sarr *et al.*, 2020 ▸; Flores-Alamo *et al.*, 2020 ▸; Lee *et al.*, 2020 ▸; de Araújo *et al.*, 2020 ▸; Prapakaran & Murugavel, 2022 ▸; Neviani *et al.*, 2022 ▸). After discarding the above-mentioned data sets, 314 data sets remained in the sample. A complete list of these with full literature references is available in the supporting information.

Statistical weights were applied in only two of the 314 analysed data sets. The fraction of systematic error in the variance of the observed intensities is 66% or more for three quarters of all data sets in the sample [Fig. 1(*a*) ▸]; for half of the data sets it is 83% or more. Either there are a lot of remaining model errors in the overwhelming majority of all published data sets in the sample or the s.u.(*I*_obs_) are flawed themselves. That underestimation of the s.u.(*I*_obs_) is a common phenomenon was also emphasized earlier (Henn & Meindl, 2015*b* ▸; Henn, 2019 ▸). Instead of ‘correcting’ underestimated s.u.(*I*_obs_) with the help of a more extensive weighting scheme, it would be more important to produce correct s.u.(*I*_obs_) in the first place. Flawed s.u.(*I*_obs_) and other systematic errors inflate the agreement factor by 3.31 times or more in 50% of the published data sets [Fig. 1(*b*) ▸].

## 4. Increase in *wR*(*F*^2^): examples from the literature

Values were taken from the literature to gain an impression of typical increases in *wR*(*F*^2^) due to systematic errors. The choice of examples is, of course, highly arbitrary, but it may still be helpful to get an impression of how much systematic errors affect the weighted agreement factor.

### 4.1. Twinning

#### 4.1.1. Non-merohedral twinning

Sevvana *et al.* (2019 ▸) discuss examples of non-merohedral twinning and give corresponding weighted agreement factors. For detailed information about the corresponding data sets, see the cited literature and the references therein. Only the results for the small-molecule data sets are discussed here. The mineral chromite, FeCr_2_O_4_ (cubic spacegroup 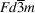), was measured on a Bruker diffractometer with Mo *K*α radiation at 292 K up to θ = 30.35°. The twin fraction was estimated to be 0.574 for the larger domain. The agreement factors for dis­regarding twinning (both domains) and for the detwinned data are compared in Table 1 ▸.

The second example is an organometallic compound 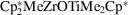 (monoclinic spacegroup *Pc*), with 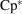 standing for pentamethylcyclopentyl, collected at 100 K with a Bruker diffractometer and Mo *K*α radiation up to θ = 25.36°.

The increase in the weighted agreement factor due to the unaccounted-for systematic error of non-merohedral twinning remains well below two in both cases. Two more examples for non-merohedral twinning were taken from the book by Müller (2006 ▸), where more information on the data sets including references is found. The first structure is diphosphonic acid, CH_6_O_6_P_2_, measured on a four-circle diffractometer with a scintillation detector (space group *P*2_1_/*c*). A large absolute off-diagonal element of 0.822 in the twin law indicates a strong overlap of the reciprocal lattices. This explains the large increase in the weighted agreement factor of 2.94 when twinning is not taken into account. The second structure, 2-(chloro­methyl)pyridinium chloride (space group *P*2_1_/*c*), was measured on a diffractometer equipped with an area detector. The twin law corresponds to a twofold rotation about one axis. Modelling of twinning does not reduce the weighted agreement factor in this case.

**Figure 2:**
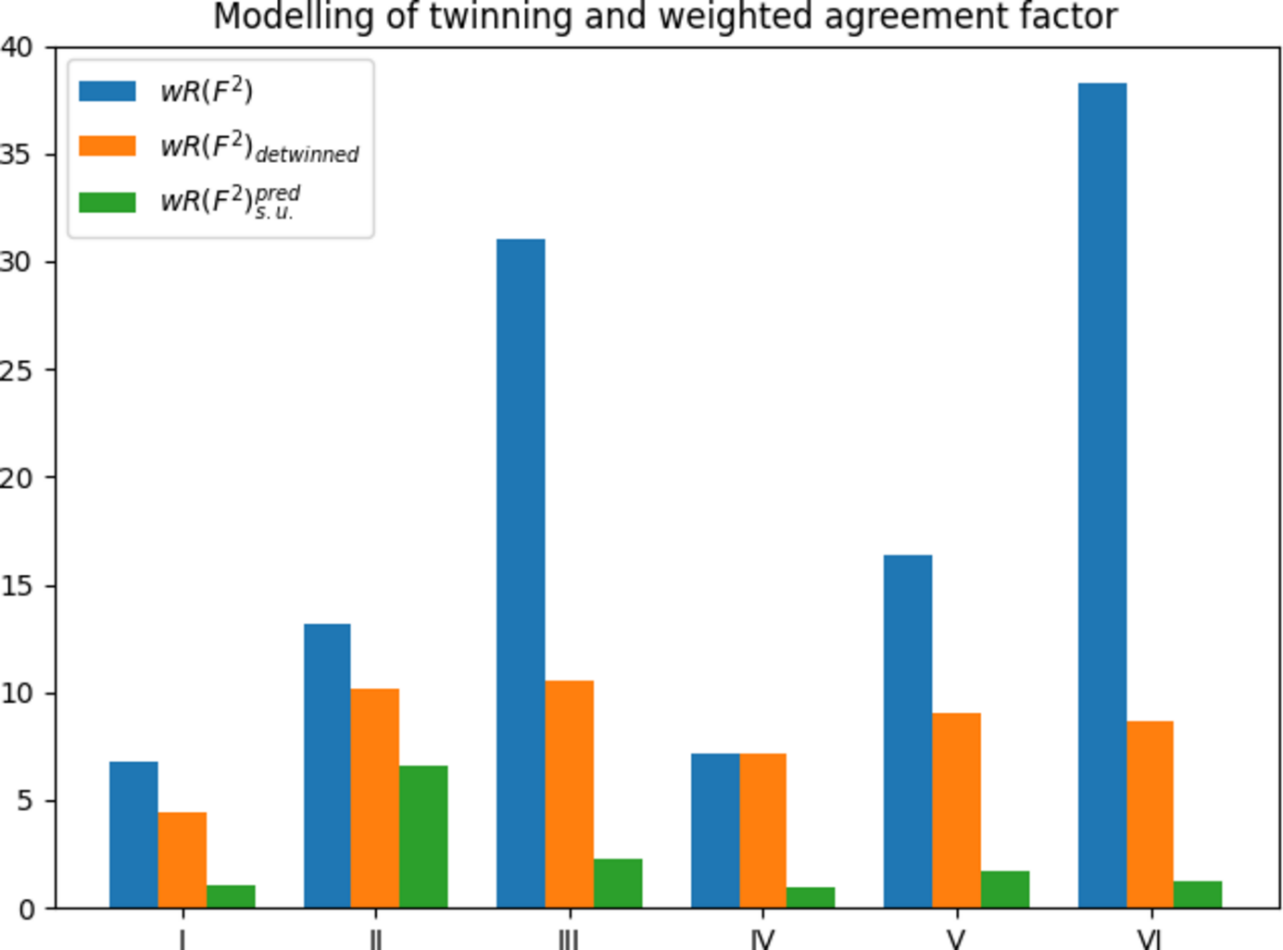
How modelling of twinning affects the weighted agreement factor. Compare with Tables 1 ▸ and 2 ▸. The blue bars show the weighted agreement prior to modelling of twinning. The orange bars show the weighted agreement factor after twinning was taken into account. Taking twinning into account reduces the weighted agreement factor in all cases, with the exception of structure IV. The green bars show the weighted agreement factor in the absence of systematic errors. The difference between the orange and green bars is the agreement factor gap. The gap is sometimes comparable to the difference between the blue and orange bars and sometimes even larger, as for structure I. The values for the agreement factors are taken from the literature (Müller, 2006 ▸) and the predicted agreement factor was calculated from the data provided therein.

In all these examples the predicted agreement factor based on σ(*I*_obs_) is smaller than the weighted agreement factor from the detwinned data sets (Fig. 2 ▸). For example, for chromite, 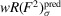 = 3.13% and the agreement factor from detwinned data *wR*(*F*^2^)_detw._ = 4.41%. The ratios 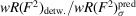 are 1.41, 1.06, 1.07 and 1.05 in the order of Table 1 ▸. These are close to one, with the exception of the chromite case in which the increase in the agreement factor due to neglect of twinning, 1.54, is similar to the increase in the weighted factor due to an unknown systematic error of 1.41. But things are even more serious when comparing the agreement factors from the detwinned data with the predicted agreement factor based on s.u.(*I*_obs_). For example, again for chromite, 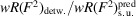 = 4.12, indicating a 4.12-fold increase in the weighted agreement factor due to other unknown systematic errors in the data set. This increase is much larger than that due to neglect of twinning (1.54). The ratios for the remaining data sets in the order of Table 1 ▸ are 1.54, 3.62 and 7.20, *i.e.* they are all larger than the corresponding reference values from detwinning.

#### 4.1.2. Obverse/reverse twins

Two structures are discussed as an example for obverse/reverse twinning; a detailed description of the structures is given by Herbst-Irmer & Sheldrick (2002 ▸). The two structures are as follows. 2,2,4,4,6,6-Hexa-*tert*-butylcyclotrisiloxane (C_24_H_54_O_3_Si_3_, trigonal space group 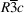, structure V) was measured on a STOE diffractometer at 133 K employing Mo *K*α radiation up to θ_max_ = 25.20°. Neglect of twinning increases the weighted agreement factor 1.82-fold. Structure VI (C_50_H_121_Al_3_F_10_Li_4_O_5_Si_9_, trigonal space group *R*3) was also measured on a STOE diffractometer, again at 133 K with Mo *K*α radiation, up to θ = 24.07°. The weighted agreement factor *wR*(*F*^2^) = 38.30% given in Table 2 ▸ corresponds to the case where twinning is not considered at all and it is compared with the case in which the obverse/reverse setting was assigned correctly [*wR*(*F*^2^) = 30.40%, not shown] and additionally with the case where merohedral twinning was taken into account, *wR*(*F*^2^) = 8.70%. This extreme case leads to a factor of 4.40 in the weighted agreement factors.

Like in the above example for non-merohedral twinning, the σ-based predicted agreement factors are close to the actual ones from the detwinned data sets. However, 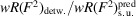 = 5.20 and 7.07. Again, an unknown systematic error leads to invoking the weighting scheme, which increases the variance in the observed intensities considerably in order to accommodate the unknown sys­tem­atic error. In other words, if there were no systematic errors in these data sets, the agreement factor would be much smaller.

### 4.2. Disorder

Some examples of relevance for disorder are discussed by Müller (2006 ▸). The individual stages in model building to solve the disorder are described in detail there, and the corresponding input and output files are also given. Some of these examples are compiled in Table 3 ▸. The agreement factors from the final stage, in which disorder is incorporated into the model, and from the initial stage are compared with each other in column 2.

**Figure 3:**
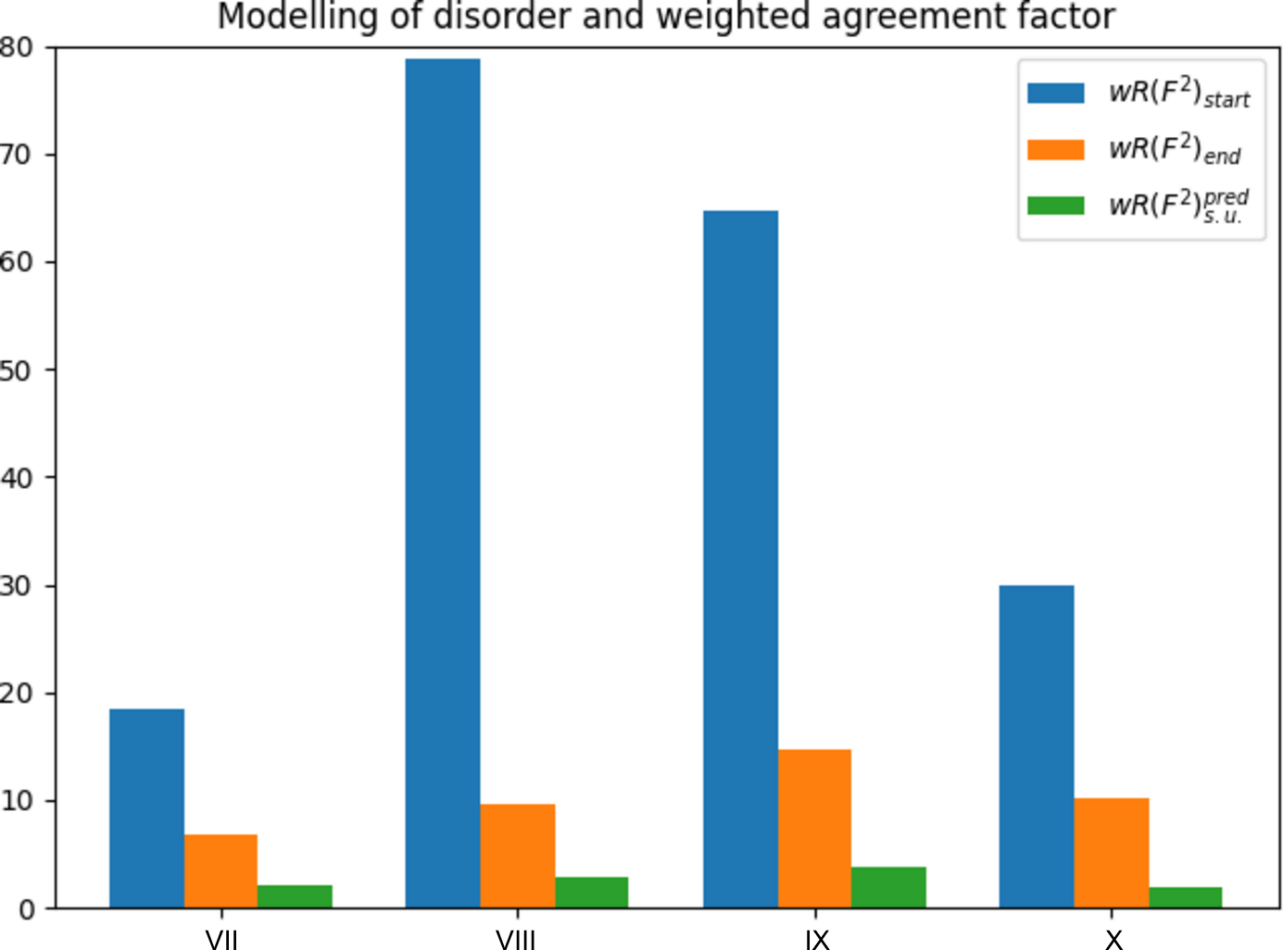
How modelling of disorder affects the weighted agreement factor. Compare with Table 3 ▸. The blue bars show the weighted agreement prior to modelling of disorder. The orange bars show the weighted agreement factor after disorder was taken into account. Taking disorder into account reduces the weighted agreement factor in all cases. The green bars show the weighted agreement factor in the absence of systematic errors. The difference between the orange and green bars is the agreement factor gap. The values for the agreement factors are taken from the literature (Müller, 2006 ▸) and the predicted agreement factor was calculated from the data provided therein.

In some cases the initial weighted agreement factor was already extremely high, like in the case of the titanium compound [*wR*(*F*^2^) = 0.7874] and in the solvent disorder case of benzoic acid [*wR*(*F*^2^) = 0.6476]. These extremely large weighted agreement factors are accompanied by the weighting scheme parameter value *a* = 0.2. It is quite rare to find such large weighting scheme parameters in published data sets. When disorder is properly accounted for, the weighted agreement factors go down by sometimes very large factors of 8.25 (Ti^III^ compound) and 4.41 (benzoic acid). The ratios of agreement factors for the remaining cases remain below three (Fig. 3 ▸).

The agreement factor ratios after taking disorder into account are 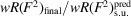 = 3.30, 3.28, 3.86 and 5.62, *i.e.* of the same order of magnitude as neglect of disorder.

### 4.3. Aspherical scattering factors and crystal environment

The values in Table 4 ▸ are taken from the article by Chodkiewicz *et al.* (2024 ▸), who apply an elaborate model called HAR± (where HAR stands for Hirshfeld atom refinement) that not only accounts for aspherical scattering factors and electron correlation at a density functional theory level of B3LYP with a rather large basis set (cc-pVTZ) but additionally takes into account to a certain degree of polarization of the molecules in the unit cell due to the crystal environment. The structure name is given in the first column. More details on these structures are found in the cited literature. The second column gives the ratio of the weighted agreement factor from an independent atom model (IAM) and from the elaborate HAR± model. Additional information about the maximum θ, wavelength λ and maximum resolution is given.

**Figure 4:**
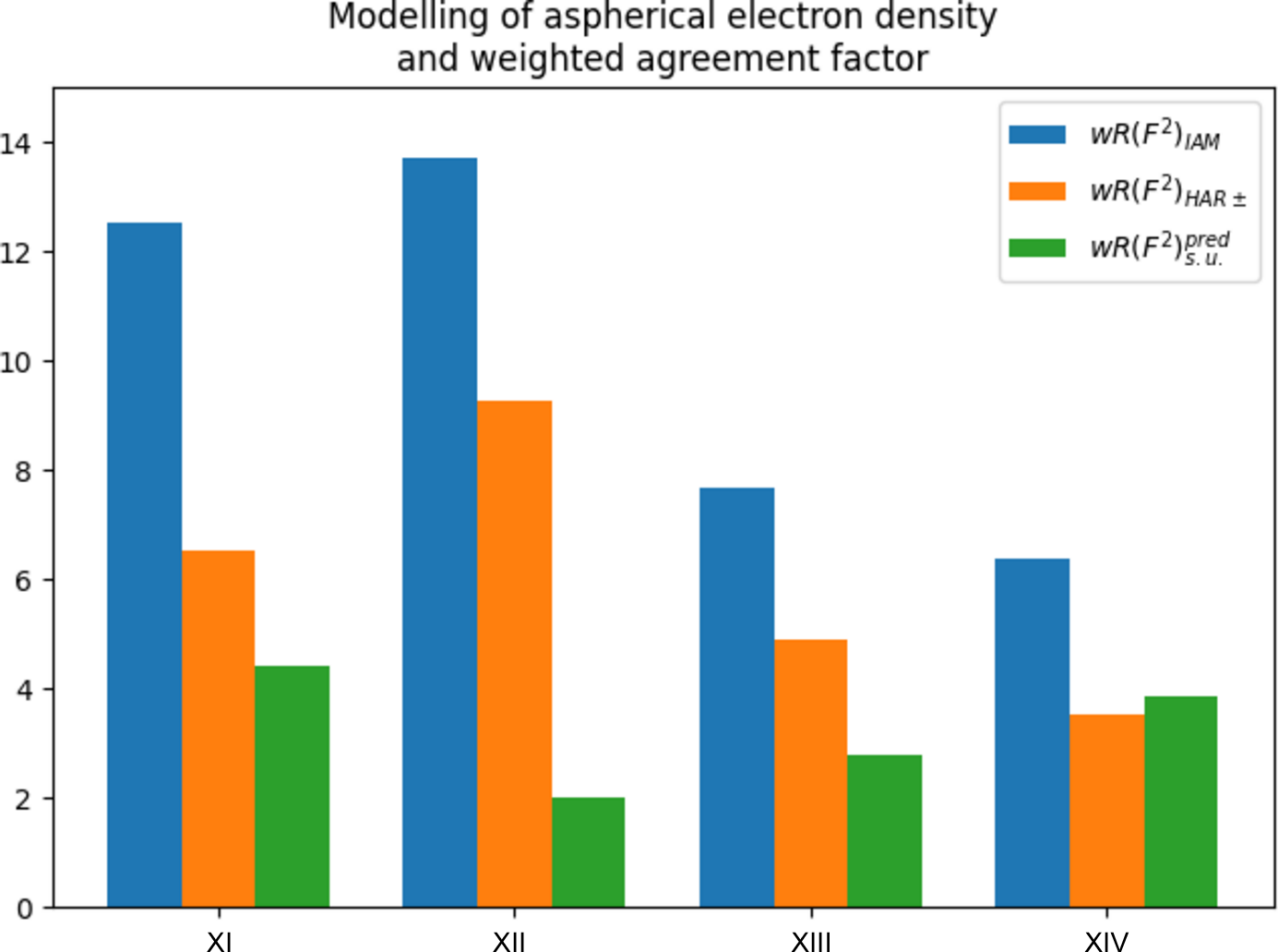
How modelling of aspherical electron density affects the weighted agreement factor. Compare with Table 4 ▸. The blue bars show the weighted agreement for the spherical independent atom model (IAM). The orange bars show the weighted agreement factor after taking aspherical effects into account. The weighted agreement factors decrease in all cases. The green bars show the weighted agreement factor in the absence of systematic errors. The difference between the orange and green bars is the agreement factor gap. The values for the agreement factors are taken from the literature [see Chodkiewicz *et al.* (2024 ▸) and references cited therein]. The predicted agreement factor was calculated from the published data.

The increase in the weighted agreement factor due to neglect of aspherical bonding density, electron correlation and polarization from the crystal field ranges between 1.48 for BIPa and 1.92 for carbamazepine, *i.e.* they are all smaller than three, even for high-resolution data sets (Fig. 4 ▸).

After taking into account bonding features and polarization of the electron density due to the crystal environment, the ratios 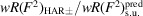 = 1.48 (carbamazepine), 4.65 (BIPa), 1.76 (NAC·H_2_O) and 0.92 (urea) indicate an unknown systematic error comparable to 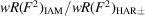 or larger in all cases but urea. The factor 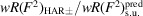 = 0.92 for urea indicates overfitting.

### 4.4. Low-energy contamination

**Figure 5:**
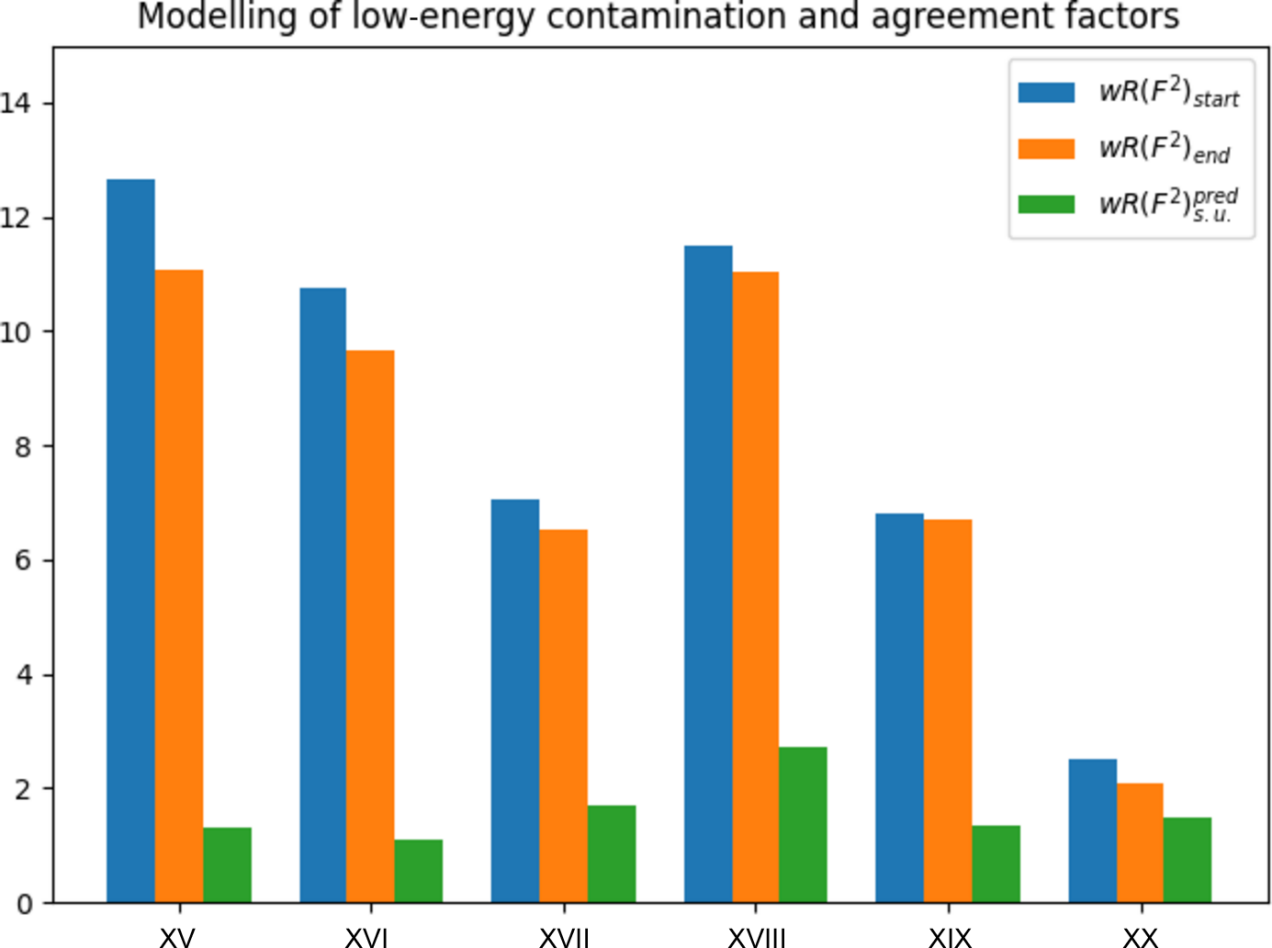
How modelling of low-energy contamination affects the weighted agreement factor. Compare with Table 5 ▸. The blue bars show the weighted agreement for the data sets contaminated by low-energy radiation. The orange bars show the weighted agreement factor after correction for low-energy contamination. The weighted agreement factors decrease in all cases. The green bars show the weighted agreement factor in the absence of systematic errors. The difference between the orange and green bars is the agreement factor gap. The respective agreement factor gap is much larger than the reduction in weighted agreement factors for structures XV–XIX. The values for the agreement factors are taken from the literature (Krause *et al.*, 2015 ▸). The predicted agreement factor was calculated from the published data.

The data in Table 5 ▸ (Fig. 5 ▸) are taken from Tables 4 and 5 of Krause *et al.* (2015 ▸) and from the corresponding CIFs. The weighted agreement factor is compared for data affected by low-energy contamination and data corrected for low-energy contamination by the empirical correction method proposed in the mentioned publication. All experimental data sets were taken on Bruker diffractometers equipped with an Incoatec microsource. Data sets XV–XVIII and XX, a high-resolution data set, were taken at 100 K, and data set XIX at 293 K. References for the data sets and more details are found in the cited literature.

The increase in the weighted agreement factor due to low-energy contamination varies between 1.02 for C_11_H_10_O_2_S (set XIX) and 1.19 for C_52_H_38_P_2_S_2_ (set XX), *i.e.* they are all much smaller than three, including the high resolution data set XX.

## 5. Discussion and conclusions

The application to published data sets in Section 3 shows that in the supposedly simple case of small-molecule crystallography – as opposed to macromolecular crystallography – systematic errors remain. These systematic errors are so large that they increase the average variance of the observed intensity and the weighted agreement factor substantially: in half of all data sets from the sample they lead to a percentage of 83% (or more) of systematic errors in the variance of the observed intensities [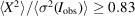] and, as a consequence, to *g* = 3.31 or more. Only 17% of the variance of the observed intensities is on average due to stochastic fluctuations as indicated by s.u.(*I*_obs_). In other words, the variance of the observed intensities as given by s.u.(*I*_obs_) is too small to explain the physical variance in the data *in virtually all data sets* from the sample. Application of a weighting scheme is needed to increase the variance to 83/17 = 4.88-fold or more in half of the data sets from the sample. This is clearly a finding that needs an explanation. Finding the correct explanation may help to reduce the agreement factor gap.

Note that the two metrics 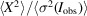 and *g* are connected to each other, as the lowest attainable weighted agreement factor is limited by the mean significance of the observed reflections. This principle is also used in the Diederichs plot (Diederichs, 2010 ▸) for data quality evaluation. An increase in the variance of the observed intensities with the help of a weighting scheme leads to a reduction in the mean significance and necessarily induces an agreement factor gap.

Different systematic errors discussed in the literature were evaluated in order to identify possible causes for the large agreement factor gap in low-resolution small-molecule crystallography and led to the following results.

Neglect of modelling of twinning in the six examples discussed led on average to a 2.17-fold increase in the weighted agreement factor. The resulting agreement factors, however, were on average still 4.70-fold increased compared with the s.u.(*I*_obs_)-based predicted agreement factor 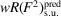; this is the value for the lowest attainable agreement factor in the absence of systematic errors with a model using the same number of model parameters *N*_par_ as in the refinement, *after modelling of twinning*. After modelling of disorder, a factor 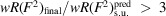 remained *in all cases*. Not modelling the asphericity of the electron density and the polarization due to the crystal environment increased the weighted agreement factor 1.69-fold on average for the discussed high-resolution data sets, but the resulting agreement factors were on average still 2.20 times larger than 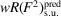 after taking asphericities and polarization of the electron densities into account.

Low-energy contamination increased the weighted agreement factor on average by 10% for the six discussed examples, but the weighted agreement factor is still *on average* 5.24 times larger than for the case without systematic errors *after correcting* for low-energy contamination. The lowest factor 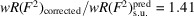 is obtained for the data set with highest resolution.

Provided the s.u.(*I*_obs_) are accurate, it remains a mystery what may cause the large median value *g* = 3.31 in the sample with *N* = 314 small-molecule data sets published in *IUCrData*. All of the above-mentioned sources of systematic errors and others may contribute. However, these discussed examples also show that *after correction* of the respective systematic errors the potential of the data as expressed by 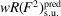 is, in virtually all cases, still not realized and therefore there is still room for progress.

A very simple and plausible interpretation of these findings is that the s.u.(*I*_obs_) are on average too small in most data sets from the sample. This hypothesis would also explain why all data sets but two employed a weighting scheme – because the s.u.(*I*_obs_) are underestimated *as standard*. Note that a *SHELXL*-like weighting scheme is not well designed to handle such an error, where the s.u.(*I*_obs_) are on average too small (Henn, 2025 ▸). It was emphasized and discussed earlier that underestimation of the s.u.(*I*_obs_), particularly of strong reflections, leads to artificially reduced weighted agreement factors [see, for example, Section 3.3 of Henn & Meindl (2015*a* ▸), Henn & Meindl (2015*b* ▸), and Sections 5 and 6 of Henn (2019 ▸)], which may unintentionally and even unknowingly pose a subliminal incentive for crystallographic software developers to rather underestimate than overestimate the s.u.(*I*_obs_) of the strong reflections. A specific systematic error in the s.u.(*I*_obs_) leading to a particular strong artificial reduction in *wR*(*F*^2^), namely underestimation of the s.u.(*I*_obs_) of the strong reflections accompanied by overestimation of the s.u.(*I*_obs_) of the weak reflections, leaves a specific trace in the weighted residuals. This trace is derived from theoretical considerations and has also been found in experimental data, as described in Sections 6.4.1 and 6.4.2 of Henn (2019 ▸). This whole discussion must be seen in the wider context of how to find accurate standard deviations of the observed intensities in diffraction experiments; this was addressed early on [see, for example, Blessing (1987 ▸)] but still appears to be unsolved.

It is concluded that there is still a problem with s.u.(*I*_obs_) that needs attention as it poses a methodological problem. For the future it is important to discriminate between those cases where a weighting scheme is applied due to flawed s.u.(*I*_obs_) and those cases where it is applied due to other model deficiencies. This will help in leading the focus back to the elimination of systematic errors and help in establishing more accurate s.u.(*I*_obs_). It will most likely also lead on average to larger agreement factors. The agreement factor gap will most likely be closed from below, by finding accurate s.u.(*I*_obs_), and from above, by eliminating or at least identifying and quantifying other remaining systematic errors.

The agreement factor gap *g* and the fraction of systematic errors in the variance of the observed intensities σ^2^(*I*_obs_) may be used as metrics for an author-based assessment of systematic errors. Providers of crystallographic data banks and publishers of crystallographic journals may apply these metrics as well and set their level of tolerance for the degree of contamination by systematic errors in submitted data sets. Quantification of the degree of contamination by systematic errors is in itself helpful for paving the way to higher data quality standards. It also shows the ‘costs’ of application of a weighting scheme in terms of the increase in the weighted agreement factor.

A threshold value may be established by using the systematic error in the variance of the observed intensities. Contamination with systematic errors less than, for example, 50% could be regarded as high quality. Implementation of these processes would entail (i) the evaluation of the degree of contamination with systematic errors and (ii) an author-based assessment of likely causes for the need to apply a weighting scheme, with the basic categories (*a*) underestimation of s.u.(*I*_obs_) and (*b*) other systematic errors with the important distinction between (*b*1) the influence of a few strong outliers on the model parameters needs to be reduced and (*b*2) a substantial part of the data (such as the weakest 10% of the intensities) show systematic differences *I*_obs_ < *I*_calc_ or *I*_obs_ > *I*_calc_ with corresponding bin mean values. Guidance from science organizing bodies such as the IUCr or from others with intrinsic motivation and interest in reducing systematic errors in deposited diffraction data like crystallographic data banks may be needed to establish such threshold values and routines. Less than 20% of the 314 data sets in the sample discussed in this work conform to this criterion of having less than 50% systematic error in the variance of the observed intensities. This is an alarming signal and calls for immediate changes, in particular since it is known that underestimation of the s.u.(*I*_obs_) of strong reflections leads to artificially lowered agreement factors and underestimation of the s.u.(*I*_obs_) of weak data leads to model bias (Henn, 2025 ▸).

## Supplementary Material

Agreement factor gaps for published data sets. DOI: 10.1107/S1600576725004376/oc5045sup1.pdf

## Figures and Tables

**Table 1:** Modelling of non-merohedral twinning Data taken from Sevvana *et al.* (2019 ▸) (structures I and II) and from the chapter *Twinning* by R. Herbst-Irmer in the book by Müller (2006 ▸) (structures III and IV).

		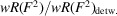	θ_max_ (°)	Wavelength (Å)	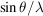 (Å^−1^)	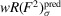 (%)	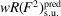 (%)	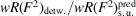
I	FeCr_2_O_4_	6.80/4.41 = 1.54	30.35	0.71073	0.71	3.13	1.07	4.12
II	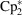 MeZrOTiMe_2_ 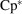	13.14/10.19 = 1.29	25.36	0.71073	0.60	9.65	6.62	1.54
III	CH_6_O_6_P_2_	31.03/10.56 = 2.94	30.06	0.71073	0.71	9.86	2.92	3.62
IV	C_6_H_7_ClN^+^·Cl^−^	7.11/7.13 = 1.00	30.47	0.71073	0.71	6.77	0.99	7.20

**Table 2:** Modelling of obverse/reverse twinning Data taken from Herbst-Irmer & Sheldrick (2002 ▸).

		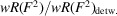	θ_max_ (°)	Wavelength (Å)	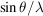 (Å^−1^)	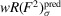 (%)	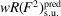 (%)	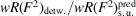
V	C_24_H_54_O_3_Si_3_	16.40/9.00 = 1.82	25.20	0.71073	0.60	8.12	1.73	5.20
VI	C_50_H_121_Al_3_F_10_Li_4_O_5_Si_9_	38.30/8.70 = 4.40	24.07	0.71073	0.57	9.21	1.23	7.07

**Table 3:** Modelling of disorder Data taken from the chapter *Disorder* by P. Müller in the book by Müller (2006 ▸).

		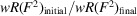	θ_max_ (°)	Wavelength (Å)	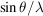 (Å^−1^)	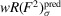 (%)	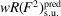 (%)	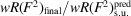
VII	Gallium iminosilicate†	18.51/6.87 = 2.69	26.37	0.71073	0.62	6.11	2.08	3.30
VIII	Ti^III^ compound‡§	78.74/9.55 = 8.25	26.99	0.71073	0.64	9.37	2.91	3.28
IX	Benzoic acid‡¶	64.76/14.69 = 4.41	54.24	1.54178	0.53	13.86	3.81	3.86
X	Toluene††	29.93/10.23 = 2.93	26.02	0.71073	0.62	8.43	1.82	5.62

† Disorder of two ethyl groups (Ga-01, Ga-06).

‡ Very large initial weighting scheme parameter *a* = 0.2.

§ Disorder of Ti^III^ cation (Ti-01, Ti-07).

¶ Disorder of a benzoic acid molecule on a twofold axis (Benz-01, Benz-04).

†† Disorder of a toluene solvent molecule about a special position (Tol-01, Tol-05).

**Table 4:** Effect of bonding density and crystal field Data taken from the article by Chodkiewicz *et al.* (2024 ▸) for results with B3LYP density functional and exponent *n* = 1. BIPa (C_25_N_11_O_16_H_25_): a co-crystal of a betaine zwitterion, two imidazolium cations and two picrate anions; NAC (C_7_H_10_NO_4_): *N*-acetyl-L-4-hydroxyproline monohydrate.

		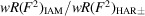	θ_max_ (°)	Wavelength (Å)	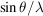 (Å^−1^)	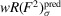 (%)	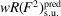 (%)	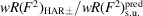
XI	Carbamazepine	12.53/6.54 = 1.92	57.99	0.71073	1.19	12.40	4.42	1.48
XII	BIPa	13.72/9.25 = 1.48	58.41	0.71073	1.20	–	1.99	4.65
XIII	NAC·H_2_O	7.67/4.90 = 1.57	31.88	0.5166	1.02	–	2.78	1.76
XIV	Urea	6.38/3.54 = 1.80	86.97	0.71073	1.41	–	3.85	0.92

**Table 5:** Effect of not-modelled low-energy contamination on *wR*(*F*^2^)

		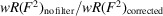	θ_max_ (°)	Wavelength (Å)	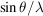 (Å^−1^)	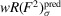 (%)	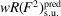 (%)	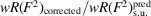
XV	C_28_H_18_N_2_	12.65/11.08 = 1.14	25.50	0.71073	0.61	11.34	1.32	8.39
XVI	C_12_H_4_N_4_	10.74/9.67 = 1.11	30.68	0.71073	0.72	9.86	1.11	8.71
XVII	C_18_H_17_CuO_6_	7.04/6.51 = 1.08	28.43	0.71073	0.67	6.59	1.68	3.88
XVIII	C_34_H_26_MgN_4_O_4_	11.48/11.02 = 1.04	30.62	0.71073	0.72	11.16	2.73	4.04
XIX	C_11_H_10_O_2_S	6.80/6.69 = 1.02	28.31	0.71073	0.67	6.25	1.33	5.03
XX	C_52_H_38_P_2_S_2_	2.50/2.10 = 1.19	52.96	0.71073	1.12	1.49	1.49	1.41

## Footnotes

1 *R*_merge_ rewards low redundancy with lower *R*_merge_, thus indicating higher accuracy, despite the accuracy of the data being expected to be lower in this case.

2 Additionally cell parameters, weighting scheme parameter values *etc.* also need to be known. However, once these are known the analysis is done with the .fcf files.
