# Supplementary material for: Two metrics for quantifying systematic errors in diffraction experiments: systematic errors in the variance of the observed intensities and agreement factor gap
Source: J Appl Crystallogr. 2025 Jun 20;58(Pt 4):1174–84. doi: 10.1107/S1600576725004376 (PMC12321034; doi:10.1107/S1600576725004376)
Supplement: Supplementary file 1 [file j-58-01174-sup1.pdf]

JOURNAL OF APPLIED CRYSTALLOGRAPHY

Volume 58 (2025)

Supporting information for article:

Two metrics for quantifying systematic errors in diffraction experiments: systematic errors in the variance of the observed intensities and agreement factor gap

Julian Henn

Journal of Applied Crystallography

ISSN 1600-5767

Two metrics for quantifying systematic errors in small molecules data sets: Agreement factor gap for small molecules, and systematic errors in the variance of the observed intensities

© 0000 International Union of Crystallography

1. Content

The supplementary material contains a table with all data sets published with IUCrData between 2020 and 2022 that were not discarded from the sample. Fist column: internal reference code; Second column: weighting scheme parameter *a*; Third column: weighting scheme parameter *b*; Fourth column: agreement factor gap *g*; Fifth column: average fraction of systematic error in the variance of the observed intensity for specified data set; Last column: Reference.

Internal ref.	a	b	g	$\frac{\langle X^2 \rangle}{\langle \sigma^2 \rangle}$	reference
2020//001	0.0197	1.6677	3.2292	0.8235	(Dean *et al.*, 2020)
2020//002	0.0991	0.6777	8.6004	0.9685	(Mamallan *et al.*, 2020)
2020//003	0.0525	2.9966	4.8220	0.9015	(Narvekar & Srinivasan, 2020*b*)
2020//004	0.0440	0.3647	2.3389	0.5977	(Morris *et al.*, 2020)
2020//005	0.0473	0.6183	6.2495	0.9678	(Yoo & Koh, 2020*b*)
2020//006	0.0734	0.2436	5.9111	0.9793	(Yoo & Koh, 2020*a*)
2020//007	0.0760	0.0000	2.4731	0.8222	(Wang, 2020)
2020//008	0.0461	1.5828	3.5354	0.5087	(Shraddha *et al.*, 2020)
2020//009	0.0511	11.2375	3.0762	0.7892	(MacNeil *et al.*, 2020)
2020//010	0.0377	0.2890	2.3051	0.6434	(Giltzau & Köckerling, 2020*a*)
2020//011	0.0178	1.2762	3.0415	0.7686	(Stammler & Imran, 2020)
2020//012	0.0065	0.5686	1.9280	0.3890	(Malin *et al.*, 2020)
2020//014	0.0390	0.5845	2.8003	0.7973	(Lough *et al.*, 2020*a*)
2020//015	0.0421	0.5355	5.1751	0.8334	(Doboszewski *et al.*, 2020)
2020//016	0.0529	0.3765	5.3220	0.9699	(Narvekar & Srinivasan, 2020*a*)
2020//017	0.0483	1.0291	3.4195	0.9276	(Weil *et al.*, 2020)
2020//018	0.1238	3.8143	11.3657	0.9967	(Manickam *et al.*, 2020)
2020//019	0.0958	0.3970	8.1871	0.9902	(Freitas *et al.*, 2020)
2020//021	0.1736	0.0000	1.7486	0.7002	(Muller II *et al.*, 2020)
2020//022	0.0424	0.1658	1.6579	0.4088	(Siegel *et al.*, 2020)
2020//023	0.0692	1.0751	4.5704	0.9552	(Powell & Rix, 2020)
2020//024	0.0349	0.0000	1.1999	0.3543	(Soundararajan *et al.*, 2020)
2020//025	0.0650	5.3126	5.0035	0.8462	(Kitada *et al.*, 2020)
2020//026	0.0426	1.6998	6.4171	0.8006	(Shahri *et al.*, 2020)
2020//027	0.2000	0.0000	18.3619	0.9989	(Rincke *et al.*, 2020)
2020//028	0.0603	1.2194	4.9950	0.9683	(Lesley *et al.*, 2020)
2020//029	0.1087	0.0972	7.7116	0.9861	(Bhat *et al.*, 2020)
2020//030	0.0554	0.0000	1.7032	0.7414	(Narayan *et al.*, 2020)
2020//031	0.0551	0.0000	6.3721	0.9506	(Detert *et al.*, 2020*b*)
2020//032	0.0567	1.3012	1.9946	0.6747	(Purdy *et al.*, 2020)
2020//033	0.0857	0.0000	4.3746	0.9671	(Tojiboev *et al.*, 2020)
2020//034	0.0478	0.6316	4.9252	0.9632	(Lough *et al.*, 2020*b*)
2020//035	0.1156	0.0000	7.1192	0.4916	(Detert *et al.*, 2020*a*)
2020//036	0.0376	0.0000	2.3519	0.8669	(Anzaldo-Olivares *et al.*, 2020)
2020//037	0.0520	0.3073	7.5641	0.9865	(Lough *et al.*, 2020*d*)
2020//038	0.0450	0.9030	4.2413	0.9389	(Lough *et al.*, 2020*c*)
2020//039	0.0717	0.7287	3.3080	0.7887	(Assoumatine & Stoeckli-Evans, 2020*b*)
2020//040	0.0237	8.9922	2.7927	0.7467	(Giltzau & Köckerling, 2020*b*)
2020//042	0.0756	0.2732	6.3209	0.9936	(Crundwell & Leeds, 2020)
2020//043	0.0736	2.1792	4.8102	0.5789	(Manjula *et al.*, 2020)
2020//044	0.0421	0.3975	4.8035	0.9514	(Chadeayne *et al.*, 2020)
2020//045	0.0750	0.0780	2.6538	0.6052	(Feightner *et al.*, 2020)
2020//046	0.0517	6.1123	2.8304	0.8679	(Carmel Y. *et al.*, 2020)
2020//047	0.0312	1.1383	1.8197	0.6059	(Dalecky *et al.*, 2020)
2020//048	0.0312	6.7359	3.3545	0.8943	(Assoumatine & Stoeckli-Evans, 2020*a*)

Internal ref.	a	b	g	$\frac{\langle X^2 \rangle}{\langle \sigma^2 \rangle}$	reference
2020//049	0.0880	32.1684	3.5853	0.7739	(Kodama *et al.*, 2020)
2020//050	0.0351	3.0856	1.5898	0.4671	(Srinivasan *et al.*, 2020*a*)
2020//051	0.0652	19.1482	5.9915	0.9450	(Geng *et al.*, 2020*b*)
2020//052	0.0141	0.0000	1.7740	0.5413	(Pérez-Benítez & Bernès, 2020)
2020//054	0.0445	0.6409	5.2586	0.9794	(Alanazi *et al.*, 2020)
2020//055	0.0797	0.0000	2.4640	0.8634	(Morales-Collazo *et al.*, 2020)
2020//056	0.0417	0.6618	5.4767	0.7077	(Cyr *et al.*, 2020)
2020//058	0.0228	0.0624	2.8129	0.6798	(Anderson *et al.*, 2020)
2020//059	0.0728	0.0917	4.3235	0.9457	(Yu *et al.*, 2020)
2020//061	0.0348	0.9424	3.1558	0.8939	(AaminaNaaz *et al.*, 2020)
2020//063	0.0655	0.2521	2.0741	0.7987	(Uzorka & LaDuca, 2020)
2020//064	0.1189	68.6175	4.7502	0.8280	(Schödel *et al.*, 2020)
2020//065	0.1069	0.0967	3.3000	0.8352	(Arunkumar *et al.*, 2020)
2020//066	0.0893	0.0000	4.1910	0.9136	(Amaro Hernández *et al.*, 2020)
2020//067	0.0380	0.0000	1.7290	0.7614	(Yoo & Koh, 2020*c*)
2020//068	0.0695	0.2851	4.7093	0.9686	(Encarnacion-Thomas *et al.*, 2020)
2020//069	0.0243	1.6832	3.5259	0.9025	(Peppel *et al.*, 2020)
2020//070	0.0208	0.9758	2.4040	0.5978	(Xu *et al.*, 2020)
2020//071	0.0255	0.6838	3.0629	0.8496	(Srinivasan *et al.*, 2020*b*)
2020//072	0.0419	0.4204	2.8508	0.8401	(Wu *et al.*, 2020)
2020//073	0.0577	2.4014	7.9499	0.8915	(Detert *et al.*, 2020*c*)
2020//074	0.1532	0.0304	5.5571	0.9617	(Shraddha & Begum, 2020)
2020//075	0.0657	0.1273	3.7461	0.9372	(Outahar *et al.*, 2020)
2020//076	0.0747	74.2699	1.7677	0.5529	(Mayorova *et al.*, 2020)
2020//078	0.0377	0.4435	3.6931	0.9332	(Devika *et al.*, 2020)
2020//079	0.0638	0.0000	2.2297	0.8312	(Linkova *et al.*, 2020)
2020//080	0.0600	0.0000	3.1707	0.5385	(Jiang *et al.*, 2020)
2020//081	0.0154	0.3678	1.4835	0.3693	(Siddiqui *et al.*, 2020)
2020//082	0.0389	1.4132	3.6061	0.9054	(Hoffman *et al.*, 2020)
2020//083	0.0210	0.7300	1.9337	0.5619	(Corfield & Stavola, 2020)
2020//084	0.0225	60.4468	3.1396	0.2088	(Marolf *et al.*, 2020)
2020//085	0.0543	6.2404	3.6194	0.8466	(Chen *et al.*, 2020*b*)
2020//086	0.1000	0.0000	2.9322	0.9330	(Lin & Li, 2020)
2020//087	0.0513	0.2183	3.3790	0.8671	(Ibragimov, 2020)
2020//088	0.0148	3.5607	2.3078	0.5002	(Deubner *et al.*, 2020)
2020//089	0.0597	0.0585	2.2343	0.7620	(Uppu *et al.*, 2020)
2020//091	0.0573	1.4452	11.0163	0.8658	(Detert & Schollmeyer, 2020*b*)
2020//092	0.0506	0.0000	1.6338	0.0171	(Shripanavar & Butcher, 2020)
2020//093	0.0000	3.3804	9.2690	0.9546	(Camargo-Cortés *et al.*, 2020)
2020//094	0.0143	16.2310	2.6356	0.6955	(Braun *et al.*, 2020)
2020//095	0.0141	2.0148	9.7152	0.8264	(Schollmeyer *et al.*, 2020)
2020//096	0.0427	1.4907	5.1279	0.9674	(Mizuhata *et al.*, 2020)
2020//097	0.0442	0.5498	4.1751	0.9345	(Whalen *et al.*, 2020)
2020//098	0.0417	0.8558	2.4765	0.8715	(Mallard *et al.*, 2020)
2020//099	0.0519	0.4095	3.3814	0.7780	(Cai *et al.*, 2020)

Internal ref.	a	b	g	$\frac{\langle X^2 \rangle}{\langle \sigma^2 \rangle}$	reference
2020//100	0.0642	0.3195	5.2367	0.9068	(Chakkarapani *et al.*, 2020)
2020//101	0.0837	0.5466	4.4379	0.9269	(Fang *et al.*, 2020)
2020//102	0.0488	0.0000	2.8920	0.9015	(Guerrero-Luna *et al.*, 2020)
2020//103	0.0611	0.0000	3.0552	0.9697	(Lynch *et al.*, 2020)
2020//104	0.0447	0.3316	3.1000	0.8908	(Chen *et al.*, 2020*a*)
2020//105	0.0466	0.6269	5.3033	0.9079	(Sung, 2020)
2020//106	0.0400	1.6585	2.8921	0.6271	(Adrian & Arman, 2020)
2020//107	0.0793	0.0000	2.3858	0.5848	(Setifi *et al.*, 2020)
2020//108	0.0684	2.1404	4.7601	0.9085	(Wang *et al.*, 2020)
2020//109	0.0375	7.1306	2.1021	0.6663	(Zometa Paniagua *et al.*, 2020)
2020//110	0.0416	0.0000	2.3689	0.8388	(Miecznikowski *et al.*, 2020)
2020//111	0.0450	0.9000	4.7529	0.9406	(Frerichs *et al.*, 2020)
2020//112	0.0357	0.0942	4.2368	0.8672	(Eckhardt *et al.*, 2020)
2020//113	0.0484	0.6202	2.6669	0.8361	(Dallasta Pedroso *et al.*, 2020)
2020//114	0.0502	0.2714	4.6232	0.9661	(Böhme & Fels, 2020)
2020//115	0.0852	0.0000	2.5403	0.8056	(Tiouabi *et al.*, 2020)
2020//116	0.0339	1.1811	6.2204	0.9452	(Kotha *et al.*, 2020*a*)
2020//117	0.0443	0.2172	4.7997	0.8482	(Padgett *et al.*, 2020)
2020//118	0.0541	1.3587	9.4040	0.8208	(Kelley *et al.*, 2020)
2020//119	0.0600	2.9000	5.2589	0.9600	(Adrian *et al.*, 2020*b*)
2020//120	0.0275	0.0000	2.1076	0.7305	(Diop *et al.*, 2020)
2020//121	0.0500	1.5000	2.8254	0.3873	(Adrian *et al.*, 2020*a*)
2020//122	0.0240	0.0000	1.8527	0.5512	(Samolová & Fábry, 2020)
2020//123	0.0253	0.0000	1.5793	0.6378	(Geng *et al.*, 2020*a*)
2020//124	0.0541	0.1743	4.1578	0.0285	(Sammeta *et al.*, 2020)
2020//125	0.0393	0.7221	1.7258	0.2881	(Kotha *et al.*, 2020*b*)
2020//126	0.0413	2.4765	6.0513	0.9615	(Harish Chinthal *et al.*, 2020)
2020//127	0.0400	0.0000	2.2905	0.7611	(Eigner, 2020)
2020//128	0.0260	0.8115	1.6161	0.4453	(Ehweiner *et al.*, 2020)
2020//129	0.0384	0.1226	4.5432	0.6617	(Böhme & Bitto, 2020)
2020//130	0.0550	0.7054	2.7606	0.8806	(Srinivasan *et al.*, 2020*c*)
2020//131	0.0237	8.5737	1.7176	0.3908	(Liang *et al.*, 2020)
2020//132	0.0568	0.9139	4.9177	0.8949	(Bendia *et al.*, 2020)
2020//133	0.0706	0.4678	7.4823	0.9832	(Nazarenko, 2020)
2020//134	0.0654	0.0319	4.0349	0.9333	(Doboszewski & Nazarenko, 2020)
2020//135	0.0379	0.0827	1.6327	0.3630	(Nagayama *et al.*, 2020)
2020//136	0.1209	3.1505	39.2595	0.9991	(Detert & Schollmeyer, 2020*a*)
2020//137	0.0583	0.3312	14.3863	0.9789	(Majer *et al.*, 2020)
2020//138	0.0408	0.2813	4.8473	0.9492	(Sha & Johnson, 2020)
2020//139	0.0556	0.0355	4.2751	0.9555	(Show *et al.*, 2020)
2020//140	0.0649	0.8212	5.1381	0.9674	(Papa *et al.*, 2020)
2021//001	0.0500	0.3528	5.5466	0.9696	(Yoo & Koh, 2021*a*)
2021//002	0.0304	0.7552	8.4197	0.7258	(Detert & Schollmeyer, 2021)
2021//003	0.0487	0.7679	4.7814	0.9078	(Chen *et al.*, 2021)

Internal ref.	a	b	g	$\frac{\langle X^2 \rangle}{\langle \sigma^2 \rangle}$	reference
2021//004	0.0751	0.4124	5.4891	0.9896	(Ovalle *et al.*, 2021)
2021//005	0.0314	0.6980	3.3152	0.9095	(Ha, 2021*d*)
2021//006	0.0152	0.8349	2.0032	0.5376	(Ha, 2021*f*)
2021//007	0.0095	1.0722	2.0352	0.4540	(Ha, 2021*e*)
2021//008	0.0258	2.4859	3.2891	0.8745	(Ha, 2021*a*)
2021//009	0.0375	5.4623	2.4729	0.7696	(Ravisankar *et al.*, 2021)
2021//010	0.0847	0.6047	3.7367	0.9574	(Saha *et al.*, 2021)
2021//011	0.0659	0.1922	3.6819	0.9610	(Vinotha *et al.*, 2021)
2021//012	0.0500	2.0000	3.7749	0.8062	(Li *et al.*, 2021)
2021//013	0.0822	2.3561	5.9657	0.6960	(Andreev *et al.*, 2021)
2021//014	0.1209	0.4577	3.3704	0.9473	(Kotha *et al.*, 2021*b*)
2021//015	0.0817	0.3242	3.4208	0.8878	(Kanagawa *et al.*, 2021)
2021//016	0.0105	0.4198	2.7963	0.7424	(Pham *et al.*, 2021)
2021//017	0.0605	0.4306	4.7235	0.9068	(Huang & Wu, 2021)
2021//018	0.0253	3.3741	2.5073	0.0762	(Al-Sudani *et al.*, 2021)
2021//019	0.0300	1.3932	3.5979	0.9030	(Ha, 2021*b*)
2021//020	0.0277	0.9047	3.7156	0.8056	(Ha, 2021*c*)
2021//021	0.0593	0.4016	2.4371	0.8563	(El-Hiti *et al.*, 2021*a*)
2021//022	0.1074	0.3866	7.3472	0.9429	(Shirmila *et al.*, 2021)
2021//023	0.0810	0.8329	2.5834	0.6327	(Reed *et al.*, 2021)
2021//024	0.0139	1.4885	1.2808	0.2640	(Schröder & Köckerling, 2021*a*)
2021//025	0.0491	0.4938	9.7943	0.9471	(Schmitt *et al.*, 2021)
2021//026	0.0331	5.8326	2.9176	0.8402	(Yan, 2021)
2021//027	0.0421	0.1915	2.3959	0.7639	(Anderson *et al.*, 2021)
2021//028	0.0296	0.5063	2.4661	0.7732	(Noland *et al.*, 2021)
2021//029	0.0739	0.6029	4.4162	0.9714	(Sathya *et al.*, 2021*b*)
2021//030	0.0812	0.2238	2.7694	0.7201	(Khanum *et al.*, 2021)
2021//031	0.0898	0.4345	3.3951	0.9282	(El-Hiti *et al.*, 2021*b*)
2021//032	0.0400	0.0000	4.7560	0.5918	(Samol̆ová *et al.*, 2021*b*)
2021//033	0.0400	0.0000	4.0935	0.7710	(Samol̆ová *et al.*, 2021*a*)
2021//034	0.0624	0.3068	2.1412	0.6054	(Kotha *et al.*, 2021*c*)
2021//035	0.0991	7.9372	3.6062	0.0469	(Ichimaru *et al.*, 2021)
2021//036	0.0816	0.0000	2.5494	0.8838	(Yang & Long, 2021)
2021//037	0.1037	0.3202	6.5899	0.9833	(Sivapriya *et al.*, 2021)
2021//038	0.0784	0.2588	9.7518	0.9865	(Mamadrakhimov *et al.*, 2021)
2021//039	0.1118	7.2076	3.9360	0.9310	(Fan & Li, 2021)
2021//040	0.0694	3.0748	4.3670	0.8876	(Sathya *et al.*, 2021*a*)
2021//041	0.0194	0.4598	3.2576	0.4995	(Bowen & Wile, 2021)
2021//042	0.0512	0.4385	3.2288	0.8791	(Menia *et al.*, 2021*a*)
2021//043	0.0606	0.5724	9.4679	0.9778	(Abou *et al.*, 2021)
2021//044	0.0494	24.3530	8.3321	0.9321	(Schollmeyer *et al.*, 2021)
2021//045	0.0506	0.2032	4.7748	0.9492	(Yoo & Koh, 2021*b*)

Internal ref.	a	b	g	$\frac{\langle X^2 \rangle}{\langle \sigma^2 \rangle}$	reference
2021//046	0.0237	2.0434	1.4904	0.4852	(Myers *et al.*, 2021)
2021//047	0.0440	0.2733	2.6035	0.8129	(Howarth *et al.*, 2021)
2021//048	0.0713	0.0000	1.6343	0.7137	(Wang *et al.*, 2021)
2021//049	0.0649	0.0000	2.2382	0.7012	(Peppel & Köckerling, 2021*b*)
2021//050	0.0674	0.1472	5.1176	0.9547	(Gao & Long, 2021)
2021//051	0.0487	0.0305	4.4501	0.9754	(Polito-Lucas *et al.*, 2021)
2021//052	0.0519	5.6305	5.5890	0.6840	(Liebing & Merzweiler, 2021)
2021//053	0.0222	1.0840	1.9486	0.6019	(Rood *et al.*, 2021)
2021//054	0.0239	0.6589	2.8780	0.8050	(Khelfa *et al.*, 2021)
2021//055	0.0664	0.1585	6.7896	0.9880	(Bernès & Hernández-Linares, 2021)
2021//056	0.0327	0.1773	3.0626	0.8450	(Thiruvalluvar *et al.*, 2021)
2021//057	0.0565	1.4766	2.3439	0.7326	(Su *et al.*, 2021)
2021//058	0.0804	1.0693	5.0823	0.9649	(Salerno *et al.*, 2021)
2021//059	0.0543	0.4521	3.7922	0.7907	(Zhang *et al.*, 2021)
2021//060	0.0910	5.3796	7.5111	0.9458	(Ivlev & Kraus, 2021)
2021//061	0.0050	1.1433	5.0337	0.6790	(Schröder & Köckerling, 2021*b*)
2021//062	0.0345	4.7462	3.0897	0.7237	(Bruekers *et al.*, 2021)
2021//063	0.0431	11.8029	7.3556	0.9120	(Harakas & Whittlesey, 2021)
2021//064	0.0247	1.1818	2.1789	0.6368	(Newman *et al.*, 2021)
2021//065	0.0282	3.0233	3.6978	0.3946	(Peppel & Köckerling, 2021*a*)
2021//066	0.0271	0.1760	1.7286	0.5714	(Rushlow *et al.*, 2021)
2021//067	0.0307	0.5240	2.6630	0.8672	(Johnston *et al.*, 2021)
2021//068	0.0578	0.6014	7.3415	0.9952	(Diop *et al.*, 2021)
2021//069	0.0616	0.7810	5.0668	0.6985	(Sung, 2021)
2021//070	0.0308	0.2127	4.1433	0.8080	(Schmidt *et al.*, 2021)
2021//071	0.0568	0.0000	2.2350	0.8187	(Guo *et al.*, 2021)
2021//072	0.0138	1.4846	1.8801	0.5937	(Yaffa *et al.*, 2021)
2021//073	0.0472	72.2358	4.6903	0.8916	(Hu *et al.*, 2021*b*)
2021//074	0.0158	0.1412	3.4532	0.8172	(Hu *et al.*, 2021*a*)
2021//076	0.0411	0.0767	3.9178	0.2439	(Olszewski & Wzgarda-Raj, 2021)
2021//077	0.0491	0.5052	4.9880	0.9616	(Taeufer *et al.*, 2021)
2021//078	0.0368	0.1955	3.6125	0.8048	(Hickstein *et al.*, 2021)
2021//079	0.0584	0.2620	2.7372	0.8724	(Patel *et al.*, 2021)
2021//080	0.0176	0.1043	1.6416	0.5623	(Hajjar *et al.*, 2021)
2021//081	0.0529	0.6752	4.2205	0.9552	(Adrian *et al.*, 2021*a*)
2021//082	0.0197	1.8079	2.2632	0.5778	(Menia *et al.*, 2021*b*)
2021//083	0.0394	0.7000	2.3877	0.8167	(Adrian *et al.*, 2021*b*)
2021//084	0.0232	1.8429	4.7294	0.2645	(Kodama *et al.*, 2021)
2021//085	0.0578	3.7997	6.1444	0.9114	(Meenatchi *et al.*, 2021)
2021//086	0.0640	0.1366	2.8231	0.5234	(Butcher & Purdy, 2021)
2021//087	0.0460	0.2103	5.3216	0.9647	(Ishida, 2021)
2021//088	0.0150	0.0500	1.4539	0.1163	(Adrian *et al.*, 2021*c*)
2021//089	0.0154	3.1892	1.8331	0.4744	(Castaldi *et al.*, 2021)
2021//090	0.0450	0.0254	3.9515	0.9513	(Lozinšek, 2021)

Internal ref.	a	b	g	$\frac{\langle X^2 \rangle}{\langle \sigma^2 \rangle}$	reference
2021//091	0.0704	0.1060	2.3888	0.7701	(Matsumoto *et al.*, 2021)
2021//092	0.0638	0.0618	2.6945	0.8286	(Fait *et al.*, 2021)
2021//093	0.0235	1.1376	2.2930	0.6489	(Kotha *et al.*, 2021*a*)
2021//094	0.0462	1.0506	2.7989	0.6854	(Pacifico & Stoeckli-Evans, 2021)
2021//095	0.0475	35.9275	4.7228	0.9122	(Davis & Muller, 2021)
2022//001	0.0758	0.8943	2.7087	0.7530	(Hatayama *et al.*, 2022)
2022//002	0.0475	0.0000	1.8983	0.3912	(Mossine *et al.*, 2022)
2022//003	0.0000	0.0000	1.1043	0.0000	(Corfield, 2022*b*)
2022//004	0.0482	0.1332	2.7837	0.9057	(Liu & Han, 2022)
2022//005	0.0463	17.4467	3.1644	0.8633	(Hu *et al.*, 2022*b*)
2022//006	0.0487	1.5639	4.5103	0.7468	(Hines III *et al.*, 2022*a*)
2022//007	0.0842	0.2113	3.3991	0.8243	(Chachlaki *et al.*, 2022*a*)
2022//008	0.1000	0.0000	4.7526	0.9870	(Surya Prakash Rao *et al.*, 2022)
2022//010	0.0953	14.3038	12.4101	0.9831	(Schollmeyer & Detert, 2022)
2022//011	0.0542	0.3200	7.2188	0.9848	(Moussa Slimane *et al.*, 2022)
2022//012	0.0524	1.0724	3.6222	0.9330	(Koehne *et al.*, 2022)
2022//013	0.0335	2.9739	2.8607	0.7717	(Hu *et al.*, 2022*a*)
2022//014	0.0335	8.3123	5.2534	0.8286	(Pacifico & Stoeckli-Evans, 2022)
2022//015	0.0624	0.0000	2.1112	0.6915	(Song *et al.*, 2022)
2022//016	0.0479	1.2289	5.6240	0.8892	(Abdallah *et al.*, 2022)
2022//017	0.0626	1.8343	4.4799	0.8633	(Meenatchi *et al.*, 2022)
2022//018	0.0409	0.6347	1.5200	0.4005	(Angevine & Benedict, 2022*a*)
2022//019	0.0000	0.2980	1.1936	0.0045	(Hines III *et al.*, 2022*b*)
2022//020	0.0231	0.0000	1.2846	0.3274	(Lambrecht *et al.*, 2022)
2022//021	0.2000	0.0000	19.0144	0.9976	(Welton *et al.*, 2022)
2022//023	0.0616	0.0000	3.3672	0.9492	(Chang, 2022)
2022//024	0.0321	0.6811	2.8324	0.8038	(Chachlaki *et al.*, 2022*b*)
2022//025	0.0183	0.0000	1.3141	0.2661	(Aquino *et al.*, 2022)
2022//026	0.0387	21.8473	3.1563	0.6828	(Yuan & Li, 2022)
2022//027	0.0167	0.8036	3.6797	0.5381	(Ghallab *et al.*, 2022*a*)
2022//028	0.0221	0.2999	3.0907	0.8611	(Ghallab *et al.*, 2022*b*)
2022//029	0.0614	0.0000	2.9541	0.8877	(Obidova *et al.*, 2022)
2022//030	0.0397	0.6757	5.9167	0.9649	(Azzam *et al.*, 2022)
2022//031	0.0204	0.5553	1.8041	0.6197	(Bosch & Bowling, 2022)
2022//032	0.0529	0.1517	3.2226	0.9117	(Pineda *et al.*, 2022)
2022//033	0.0473	0.2757	5.6415	0.9634	(Naeem *et al.*, 2022)
2022//034	0.0686	10.1161	4.1444	0.8921	(Cao *et al.*, 2022)
2022//035	0.0438	5.3510	2.4820	0.6999	(Deng *et al.*, 2022)
2022//036	0.0464	1.8748	4.9080	0.7415	(Neal *et al.*, 2022)
2022//037	0.0076	10.8403	3.5966	0.4636	(Schuster *et al.*, 2022)
2022//038	0.0115	1.2666	2.0347	0.4854	(Smith *et al.*, 2022)
2022//039	0.0606	0.4983	2.3899	0.8061	(Angevine & Benedict, 2022*b*)
2022//040	0.0492	3.5802	4.9639	0.7549	(Bruekers *et al.*, 2022)
2022//041	^{0}.0009	1.6438	1.2279	0.0834	(Franklin *et al.*, 2022)
2022//042	0.0689	0.1685	4.6781	0.9452	(Guerrab *et al.*, 2022)

Internal ref.	a	b	g	$\frac{\langle X^2 \rangle}{\langle \sigma^2 \rangle}$	reference
2022//043	0.0409	0.0000	1.3355	0.5733	(Brihi *et al.*, 2022)
2022//044	0.0899	0.4855	4.6742	0.9582	(Zaoui *et al.*, 2022)
2022//045	0.0523	0.0000	1.9121	0.8852	(Pérez-Benítez *et al.*, 2022)
2022//046	0.0359	173.2937	3.9830	0.8582	(Schröder & Köckerling, 2022)
2022//047	0.0000	0.0000	0.9176	0.0000	(Corfield, 2022*a*)
2022//048	0.0988	1.2801	4.1406	0.9629	(Missioui *et al.*, 2022)
2022//049	0.0384	1.2273	2.8673	0.6670	(Abad *et al.*, 2022)
2022//050	0.0234	23.3944	2.6287	0.7725	(Rushlow *et al.*, 2022)
2022//051	0.0583	1.8772	3.2617	0.6677	(Moris & Galdámez, 2022)
2022//052	0.0431	226.7933	3.3012	0.8497	(Zhou *et al.*, 2022)
2022//054	0.0622	0.1317	2.3415	0.7791	(Shank *et al.*, 2022)
2022//055	0.0317	0.0410	2.0678	0.7640	(Newell *et al.*, 2022)
2022//056	0.1278	0.0000	2.0962	0.8070	(Ichimaru *et al.*, 2022)
2022//057	0.0255	0.9013	4.6306	0.8251	(Harakas & Demmin, 2022)
2022//058	0.0194	21.8494	2.5887	0.5885	(Shevlin *et al.*, 2022)
2022//059	0.0336	4.2080	2.5082	0.6891	(Ren *et al.*, 2022)
2022//060	0.0425	5.6399	3.1925	0.8140	(Potgieter *et al.*, 2022*a*)
2022//061	0.0747	4.1236	3.0545	0.8654	(Potgieter *et al.*, 2022*c*)
2022//062	0.0537	0.2098	4.3211	0.9065	(Okuno & Doi, 2022)
2022//063	0.0514	2.0580	3.4048	0.6934	(Koh, 2022)
2022//064	0.0826	0.0000	4.5747	0.9806	(Martínez-de la Luz *et al.*, 2022)
2022//065	0.0554	0.2283	2.0366	0.4747	(Archana *et al.*, 2022*a*)
2022//066	0.0372	0.0000	3.1987	0.6548	(Priyanka *et al.*, 2022)
2022//067	0.0727	0.8943	4.7550	0.9576	(Sung, 2022)
2022//068	0.0133	5.3948	2.4597	0.6331	(Blackwelder *et al.*, 2022)
2022//069	0.0532	9.0949	1.9826	0.3142	(Shimazaki & Sadakiyo, 2022*a*)
2022//070	0.0390	0.1001	2.6237	0.7299	(Bellia *et al.*, 2022)
2022//071	0.0273	0.6595	1.5394	0.4446	(Ren & Li, 2022)
2022//073	0.0646	0.4782	3.8041	0.9502	(Saini *et al.*, 2022)
2022//074	0.0555	0.4922	2.6745	0.8131	(Archana *et al.*, 2022*b*)
2022//075	0.0371	2.3114	5.2169	0.8376	(Campos Fernandez *et al.*, 2022)
2022//076	0.0555	0.6357	1.6254	0.4006	(Shimazaki & Sadakiyo, 2022*b*)
2022//077	0.0642	0.1082	2.7387	0.6674	(Vinaya *et al.*, 2022)
2022//078	0.0230	0.8521	1.6761	0.3620	(Sánchez-García *et al.*, 2022)
2022//079	0.0845	0.0881	4.0066	0.8680	(Khan *et al.*, 2022)
2022//080	0.0685	0.8521	3.3130	0.8817	(Archana *et al.*, 2022*c*)
2022//081	0.0515	0.0000	2.4009	0.5901	(Butcher & Purdy, 2022*b*)
2022//082	0.0510	1.0570	5.4016	0.9603	(Mohamed-Ezzat *et al.*, 2022)
2022//083	0.0100	25.0000	7.3501	0.9543	(Adrian *et al.*, 2022*b*)
2022//084	0.0290	20.5034	2.9338	0.5703	(Malan *et al.*, 2022*a*)
2022//085	0.0587	0.3308	10.9288	0.9003	(Halz *et al.*, 2022)
2022//086	0.0449	0.2210	3.7380	0.7835	(Lutz & Kroon-Batenburg, 2022)
2022//087	0.0166	0.2838	2.3417	0.5341	(Butcher & Purdy, 2022*a*)
2022//088	0.0294	0.4397	3.2430	0.6521	(Saadallah *et al.*, 2022)
2022//089	0.0164	1.1947	1.9685	0.3305	(Graaff *et al.*, 2022)
2022//090	0.0325	2.6340	2.7890	0.8469	(Malan *et al.*, 2022*b*)
2022//091	0.0341	5.9508	2.9120	0.7678	(Potgieter *et al.*, 2022*b*)
2022//092	0.0506	0.7400	2.6392	0.8257	(Adrian *et al.*, 2022*c*)
2022//093	0.0618	0.5328	2.8688	0.8629	(Adrian *et al.*, 2022*a*)

Acknowledgements

References

AaminaNaaz, Y., Rajkumar, K., Thirumurugan, S., Anbalagan, K. & SubbiahPandi, A. (2020). *IUCrData*, **5**(5), x200618. https://doi.org/10.1107/S2414314620006185

Abad, N., Missioui, M., Alsubari, A., Mague, J. T., Essassi, E. M. & Ramli, Y. (2022). *IUCrData*, **7**(7). https://doi.org/10.1107/S2414314622006939

Abdallah, A. E. M., Elgemeie, G. H. & Jones, P. G. (2022). *IUCrData*, **7**(3), x220332. https://doi.org/10.1107/S2414314622003327

Abou, A., Bamba, F., Marrot, J., Yaya, S. & Coustard, J.-M. (2021). *IUCrData*, **6**(6), x210674. https://doi.org/10.1107/S241431462100674X

Adrian, R. A. & Arman, H. D. (2020). *IUCrData*, **5**(9), x201292. https://doi.org/10.1107/S2414314620012924

Adrian, R. A., Canales, D. & Arman, H. D. (2020*a*). *IUCrData*, **5**(10), x201344. https://doi.org/10.1107/S2414314620013449

Adrian, R. A., Duarte, J. J. & Arman, H. D. (2021*a*). *IUCrData*, **6**(10), x211096. https://doi.org/10.1107/S2414314621010968

Adrian, R. A., Gutierrez, M. C. & Arman, H. D. (2022*a*). *IUCrData*, **7**(12), x221151. https://doi.org/10.1107/S2414314622011518

Adrian, R. A., Hernandez, D. R. & Arman, H. D. (2020*b*). *IUCrData*, **5**(10), x201407. https://doi.org/10.1107/S2414314620014078

Adrian, R. A., Ibarra, S. J. & Arman, H. D. (2021*b*). *IUCrData*, **6**(10), x211073. https://doi.org/10.1107/S2414314621010737

Adrian, R. A., Ibarra, S. J. & Arman, H. D. (2022*b*). *IUCrData*, **7**(11), x221096. https://doi.org/10.1107/S2414314622010963

Adrian, R. A., Lagemann, B. J. & Arman, H. D. (2021*c*). *IUCrData*, **6**(11), x211171. https://doi.org/10.1107/S2414314621011718

Adrian, R. A., Rios, J. J. & Arman, H. D. (2022*c*). *IUCrData*, **7**(12), x221149. https://doi.org/10.1107/S241431462201149X

Al-Sudani, A.-R. H., Abdulridha, M. Q. & Kariuki, B. M. (2021). *IUCrData*, **6**(2), x210150. https://doi.org/10.1107/S2414314621001504

Alanazi, S. A., Abdel-Wahab, B. F., Hegazy, A. S., Kariuki, B. M. & El-Hiti, G. A. (2020). *IUCrData*, **5**(5), x200700. https://doi.org/10.1107/S2414314620007002

Amaro Hernández, A. G., Rodríguez Tzompantzi, T., Dávila García, Á., Meza-León, R. L. & Bernès, S. (2020). *IUCrData*, **5**(6), x200788. https://doi.org/10.1107/S2414314620007889

Anderson, G., Mirjafari, A., Zeller, M. & Hillesheim, P. C. (2020). *IUCrData*, **5**(5), x200660. https://doi.org/10.1107/S2414314620006604

Anderson, G. I., Bellia, S., Zeller, M., Hillesheim, P. C. & Mirjafari, A. (2021). *IUCrData*, **6**(4), x210406. https://doi.org/10.1107/S2414314621004065

Andreev, S., Schollmeyer, D. & Koch, P. (2021). *IUCrData*, **6**(2), x210159. https://doi.org/10.1107/S2414314621001590

Angevine, D. J. & Benedict, J. B. (2022*a*). *IUCrData*, **7**(3), x220304. https://doi.org/10.1107/S2414314622003042

Angevine, D. J. & Benedict, J. B. (2022*b*). *IUCrData*, **7**(5), x220510. https://doi.org/10.1107/S2414314622005107

Anzaldo-Olivares, B., Arroyo, M., Ramírez-Monroy, A. & Bernès, S. (2020). *IUCrData*, **5**(3), x200281. https://doi.org/10.1107/S2414314620002813

Aquino, A. J., Gerrior, D., Cameron, T. S., Robertson, K. N. & Aquino, M. A. (2022). *IUCrData*, **7**(3), x220249. https://doi.org/10.1107/S2414314622002498

Archana, S. D., Nagma Banu, H. A., Kalluraya, B., Yathirajan, H. S., Balerao, R. & Butcher, R. J. (2022*a*). *IUCrData*, **7**(9), x220924. https://doi.org/10.1107/S2414314622009245

Archana, S. D., Nagma Banu, H. A., Kalluraya, B., Yathirajan, H. S., Balerao, R. & Butcher, R. J. (2022*b*). *IUCrData*, **7**(10), x221003. https://doi.org/10.1107/S2414314622010033

Archana, S. D., Nagma Banu, H. A., Kalluraya, B., Yathirajan, H. S., Balerao, R. & Butcher, R. J. (2022*c*). *IUCrData*, **7**(11), x221047. https://doi.org/10.1107/S2414314622010471

Arunkumar, D., Samshuddin, S., Ansar, M., Mague, J. T. & Ramli, Y. (2020). *IUCrData*, **5**(6), x200800. https://doi.org/10.1107/S2414314620008007

Assoumatine, T. & Stoeckli-Evans, H. (2020*a*). *IUCrData*, **5**(4), x200467. https://doi.org/10.1107/S2414314620004678

Assoumatine, T. & Stoeckli-Evans, H. (2020*b*). *IUCrData*, **5**(3), x200401. https://doi.org/10.1107/S2414314620004010

Azzam, R. A., Elgemeie, G. H., Gad, N. M. & Jones, P. G. (2022). *IUCrData*, **7**(4), x220412. https://doi.org/10.1107/S2414314622004126

Bellia, S., Anderson, G., Zeller, M., Mirjafari, A. & Hillesheim, P. C. (2022). *IUCrData*, **7**(9), x220878. https://doi.org/10.1107/S2414314622008781

Bendia, S., Ouari, K., Ait Ali, M., Firdousi, L. e. & Nazarenko, A. Y. (2020). *IUCrData*, **5**(12), x201662. https://doi.org/10.1107/S2414314620016624

Bernès, S. & Hernández-Linares, M. G. (2021). *IUCrData*, **6**(7), x210697. https://doi.org/10.1107/S2414314621006970

Bhat, R., Shraddha, K. N. & Begum, N. S. (2020). *IUCrData*, **5**(3), x200385. https://doi.org/10.1107/S2414314620003855

Blackwelder, L. A., Kelley, A. R., Balaich, G. J. & Jefferies, L. R. (2022). *IUCrData*, **7**(9), x220895. https://doi.org/10.1107/S2414314622008951

Böhme, U. & Bitto, F. (2020). *IUCrData*, **5**(11), x201444. https://doi.org/10.1107/S2414314620014443

Böhme, U. & Fels, S. (2020). *IUCrData*, **5**(10), x201384. https://doi.org/10.1107/S241431462001384X

Bosch, E. & Bowling, N. P. (2022). *IUCrData*, **7**(4), x220380. https://doi.org/10.1107/S2414314622003807

Bowen, C. L. & Wile, B. M. (2021). *IUCrData*, **6**(5), x210516. https://doi.org/10.1107/S2414314621005162

Braun, J. D., Uppal, G. & Herbert, D. E. (2020). *IUCrData*, **5**(8), x201048. https://doi.org/10.1107/S2414314620010482

Brihi, O., Medjani, M., Bougueria, H., Djedouani, A., Francois, M., Fleutot, S. & Boudjada, A. (2022). *IUCrData*, **7**(6), x220577. https://doi.org/10.1107/S2414314622005776

Bruekers, J., Elemans, J., Nolte, R. & Tinnemans, P. (2021). *IUCrData*, **6**(8), x210768. https://doi.org/10.1107/S2414314621007689

Bruekers, J. P. J., Elemans, J. A. A. W., Nolte, R. J. M. & Tinnemans, P. (2022). *IUCrData*, **7**(5), x220489. https://doi.org/10.1107/S2414314622004898

Butcher, R. J. & Purdy, A. P. (2021). *IUCrData*, **6**(11), x211098. https://doi.org/10.1107/S2414314621010981

Butcher, R. J. & Purdy, A. P. (2022*a*). *IUCrData*, **7**(12), x221172. https://doi.org/10.1107/S2414314622011725

Butcher, R. J. & Purdy, A. P. (2022*b*). *IUCrData*, **7**(11), x221049. https://doi.org/10.1107/S2414314622010495

Cai, C., Lin, B., Wu, W. & Zhu, Q. (2020). *IUCrData*, **5**(9), x201246. https://doi.org/10.1107/S2414314620012468

Camargo-Cortés, E. B., Acosta, M., Martínez, J. C. & Pineda, L. W. (2020). *IUCrData*, **5**(8), x201092. https://doi.org/10.1107/S2414314620010925

Campos Fernandez, C., Procupez-Schtirbu, R., Soto-Tellini, V. H., Salazar, J. C. & Jancik, V. (2022). *IUCrData*, **7**(10), x220947. https://doi.org/10.1107/S2414314622009476

Cao, H., Wang, J. & Li, J. (2022). *IUCrData*, **7**(4), x220386. https://doi.org/10.1107/S2414314622003868
Carmel Y., S., Prasad, N. L., Begum, N. S. & Suresh, H. P. (2020). *IUCrData*, **5**(4), x200427. https://doi.org/10.1107/S2414314620004277
Castaldi, K. T., Astashkin, A. V., Albert, D. R. & Rajaseelan, E. (2021). *IUCrData*, **6**(11), x211142. https://doi.org/10.1107/S2414314621011421
Chachlaki, E., Choquesillo-Lazarte, D. & Demadis, K. D. (2022*a*). *IUCrData*, **7**(2), x220189. https://doi.org/10.1107/S2414314622001894
Chachlaki, E., Choquesillo-Lazarte, D. & Demadis, K. D. (2022*b*). *IUCrData*, **7**(3), x220247. https://doi.org/10.1107/S2414314622002474
Chadeayne, A. R., Pham, D. N. K., Golen, J. A. & Manke, D. R. (2020). *IUCrData*, **5**(4), x200498. https://doi.org/10.1107/S2414314620004988
Chakkarapani, N., Murugan, S., Ibrahim, A. R., Kavitha, S. J., Hemamalini, M. & Rajakannan, V. (2020). *IUCrData*, **5**(9), x201239. https://doi.org/10.1107/S2414314620012390
Chang, J.-G. (2022). *IUCrData*, **7**(3), x220295. https://doi.org/10.1107/S2414314622002954
Chen, L., Hu, J. & Sun, H.-S. (2021). *IUCrData*, **6**(1), x210018. https://doi.org/10.1107/S2414314621000183
Chen, L., Hu, J., Wu, L.-L. & Sun, H.-S. (2020*a*). *IUCrData*, **5**(9), x201213. https://doi.org/10.1107/S2414314620012134
Chen, Y., Wang, B., Fontenot, P. & Donahue, J. P. (2020*b*). *IUCrData*, **5**(7), x200939. https://doi.org/10.1107/S2414314620009396
Corfield, P. W. R. (2022*a*). *IUCrData*, **7**(6), x220607. https://doi.org/10.1107/S2414314622006071
Corfield, P. W. R. (2022*b*). *IUCrData*, **7**(1), x211351. https://doi.org/10.1107/S2414314621013511
Corfield, P. W. R. & Stavola, T. J. (2020). *IUCrData*, **5**(7), x200968. https://doi.org/10.1107/S2414314620009682
Crundwell, G. & Leeds, A. (2020). *IUCrData*, **5**(4), x200454. https://doi.org/10.1107/S241431462000454X
Cyr, N., Zeller, M., Hillesheim, P. C. & Mirjafari, A. (2020). *IUCrData*, **5**(5), x200689. https://doi.org/10.1107/S2414314620006896
Dalecky, L. M., Juillerat, C. A. & Cody, J. A. (2020). *IUCrData*, **5**(4), x200312. https://doi.org/10.1107/S2414314620003120
Dallasta Pedroso, S., Caracelli, I., Zukerman-Schpector, J., Soto-Monsalve, M., De Almeida Santos, R. H., Correia, C. R. D., Llanes Garcia, A. L. & Tiekink, E. R. T. (2020). *IUCrData*, **5**(10), x201228. https://doi.org/10.1107/S2414314620012286
Davis, W. L. & Muller, A. (2021). *IUCrData*, **6**(12), x211259. https://doi.org/10.1107/S2414314621012591
Dean, R., Miller, C. N., Zingales, S. K. & Padgett, C. W. (2020). *IUCrData*, **5**(1), x200110. https://doi.org/10.1107/S2414314620001108
Deng, M., Yin, Y., Wang, S.-S., Qi, X.-Y. & Zhu, A.-X. (2022). *IUCrData*, **7**(4), x220377. https://doi.org/10.1107/S2414314622003777
Detert, H., Jacobs, N. & Schollmeyer, D. (2020*a*). *IUCrData*, **5**(3), x200307. https://doi.org/10.1107/S2414314620003077
Detert, H., Jochem, M. & Schollmeyer, D. (2020*b*). *IUCrData*, **5**(3), x200372. https://doi.org/10.1107/S2414314620003727
Detert, H., Kluge, L. & Schollmeyer, D. (2020*c*). *IUCrData*, **5**(7), x201018. https://doi.org/10.1107/S2414314620010184
Detert, H. & Schollmeyer, D. (2020*a*). *IUCrData*, **5**(12), x201585. https://doi.org/10.1107/S2414314620015850
Detert, H. & Schollmeyer, D. (2020*b*). *IUCrData*, **5**(8), x201081. https://doi.org/10.1107/S2414314620010810
Detert, H. & Schollmeyer, D. (2021). *IUCrData*, **6**(1), x210069. https://doi.org/10.1107/S2414314621000699
Deubner, H. L., Ivlev, S. I. & Kraus, F. (2020). *IUCrData*, **5**(7), x200960. https://doi.org/10.1107/S2414314620009608
Devika, S., Begum, N. S., Manjappa, K. B. & Yang, D.-Y. (2020). *IUCrData*, **5**(7), x200857. https://doi.org/10.1107/S2414314620008573
Diop, A., Diop, T., Diop, C. A. K., Tumanov, N., Ennio, Z. & Sidibé, M. (2021). *IUCrData*, **6**(9), x210989. https://doi.org/10.1107/S2414314621009895
Diop, A., Diop, T., Kama, A. B., Diop, C. A. K. & Tumanov, N. (2020). *IUCrData*, **5**(10), x201423. https://doi.org/10.1107/S2414314620014236
Doboszewski, B. & Nazarenko, A. Y. (2020). *IUCrData*, **5**(12), x201630. https://doi.org/10.1107/S2414314620016302
Doboszewski, B., Nazarenko, A. Y. & Soares, F. d. P. (2020). *IUCrData*, **5**(2), x200257. https://doi.org/10.1107/S2414314620002576
Eckhardt, T., Wagner, C., Imming, P. & Seidel, R. W. (2020). *IUCrData*, **5**(9), x201275. https://doi.org/10.1107/S2414314620012754
Ehweiner, M. A., Belaj, F. & Mösch-Zanetti, N. C. (2020). *IUCrData*, **5**(11), x201465. https://doi.org/10.1107/S2414314620014650
Eigner, V. (2020). *IUCrData*, **5**(11), x201511. https://doi.org/10.1107/S2414314620015114
El-Hiti, G. A., Abdel-Wahab, B. F., Baashen, M. A., Yousif, E., Hegazy, A. S. & Kariuki, B. M. (2021*a*). *IUCrData*, **6**(3), x210314. https://doi.org/10.1107/S241431462100314X
El-Hiti, G. A., Abdel-Wahab, B. F., Yousif, E., Hegazy, A. S. & Kariuki, B. M. (2021*b*). *IUCrData*, **6**(4), x210318. https://doi.org/10.1107/S2414314621003187
Encarnacion-Thomas, E., Sommer, R. D., Mallia, A. & Sloop, J. (2020). *IUCrData*, **5**(6), x200759. https://doi.org/10.1107/S2414314620007592
Fait, M. J. G., Spannenberg, A., Kondratenko, E. V. & Linke, D. (2021). *IUCrData*, **6**(12), x211332. https://doi.org/10.1107/S2414314621013328
Fan, Y. & Li, J. (2021). *IUCrData*, **6**(5). https://doi.org/10.1107/S2414314621005319
Fang, Y., Liu, B. & Jia, Z. (2020). *IUCrData*, **5**(9), x201241. https://doi.org/10.1107/S2414314620012419
Feightner, K., Powell, D. R. & Burba, C. M. (2020). *IUCrData*, **5**(4), x200445. https://doi.org/10.1107/S2414314620004459
Franklin, D., Lee, A., Fronczek, F. R. & Junk, T. (2022). *IUCrData*, **7**(6), x220622. https://doi.org/10.1107/S2414314622006228
Freitas, J. F. d., Brown, S., Oberndorfer, J. S. & Crundwell, G. (2020). *IUCrData*, **5**(2), x200203. https://doi.org/10.1107/S2414314620002035
Frerichs, N., Schmidtmann, M. & Beckhaus, R. (2020). *IUCrData*, **5**(9), x201145. https://doi.org/10.1107/S2414314620011451
Gao, J. & Long, S. (2021). *IUCrData*, **6**(6), x210555. https://doi.org/10.1107/S2414314621005551
Geng, X., Wen, B. & Fan, C. (2020*a*). *IUCrData*, **5**(10), x201412. https://doi.org/10.1107/S2414314620014121
Geng, X., Wen, B. & Fan, C. (2020*b*). *IUCrData*, **5**(4), x200422. https://doi.org/10.1107/S2414314620004228
Ghallab, R., Bougueria, H. & Merazig, H. (2022*a*). *IUCrData*, **7**(3), x220195. https://doi.org/10.1107/S241431462200195X
Ghallab, R., Bougueria, H. & Merazig, H. (2022*b*). *IUCrData*, **7**(3), x220191. https://doi.org/10.1107/S2414314622001912
Giltzau, N. O. & Köckerling, M. (2020*a*). *IUCrData*, **5**(1), x200023. https://doi.org/10.1107/S2414314620000231

Giltzau, N. O. & Köckerling, M. (2020*b*). *IUCrData*, **5**(3), x200261. https://doi.org/10.1107/S2414314620002618

Graaff, S. d., Elma, A., Schmidtmann, M. & Beckhaus, R. (2022). *IUCrData*, **7**(12), x221201. https://doi.org/10.1107/S2414314622012019

Guerrab, W., El Moutaouakil Ala Allah, A., Alsubari, A., Mague, J. T. & Ramli, Y. (2022). *IUCrData*, **7**(6), x220598. https://doi.org/10.1107/S2414314622005983

Guerrero-Luna, G., Reyes Melchor, J., Bernès, S. & Hernández-Linares, M.-G. (2020). *IUCrData*, **5**(9), x201200. https://doi.org/10.1107/S2414314620012006

Guo, H., Du, W. & Zhou, H. (2021). *IUCrData*, **6**(9), x210901. https://doi.org/10.1107/S2414314621009019

Ha, K. (2021*a*). *IUCrData*, **6**(1), x210084. https://doi.org/10.1107/S2414314621000845

Ha, K. (2021*b*). *IUCrData*, **6**(2), x210153. https://doi.org/10.1107/S241431462100153X

Ha, K. (2021*c*). *IUCrData*, **6**(2), x210094. https://doi.org/10.1107/S2414314621000948

Ha, K. (2021*d*). *IUCrData*, **6**(1), x210093. https://doi.org/10.1107/S2414314621000936

Ha, K. (2021*e*). *IUCrData*, **6**(1), x210083. https://doi.org/10.1107/S2414314621000833

Ha, K. (2021*f*). *IUCrData*, **6**(1), x210085. https://doi.org/10.1107/S2414314621000857

Hajjar, C., Ovens, J. S. & Bryce, D. L. (2021). *IUCrData*, **6**(10), x211044. https://doi.org/10.1107/S2414314621010440

Halz, J. H., Heiser, C. & Merzweiler, K. (2022). *IUCrData*, **7**(11), x221035. https://doi.org/10.1107/S2414314622010355

Harakas, G. N. & Demmin, M. E. (2022). *IUCrData*, **7**(8), x220832. https://doi.org/10.1107/S241431462200832X

Harakas, G. N. & Whittlesey, B. R. (2021). *IUCrData*, **6**(8), x210845. https://doi.org/10.1107/S2414314621008452

Harish Chinthal, C., Kavitha, C. N., Yathirajan, H. S., Foro, S. & Glidewell, C. (2020). *IUCrData*, **5**(11), x201523. https://doi.org/10.1107/S2414314620015230

Hatayama, Y., Akagi, K. & Okuno, T. (2022). *IUCrData*, **7**(1), x220083. https://doi.org/10.1107/S2414314622000839

Hickstein, D. D., Reinheimer, E. W., Johnson, A. R. & O'Leary, D. J. (2021). *IUCrData*, **6**(10), x211043. https://doi.org/10.1107/S2414314621010439

Hines III, J. E., Deere, C. J., Fronczek, F. R. & Uppu, R. M. (2022*a*). *IUCrData*, **7**(2), x220201. https://doi.org/10.1107/S2414314622002012

Hines III, J. E., Deere, C. J., Vaddi, P., Kondati, R. R., Fronczek, F. R. & Uppu, R. M. (2022*b*). *IUCrData*, **7**(3), x220277. https://doi.org/10.1107/S2414314622002772

Hoffman, J. L., Akhigbe, J. E., Reinheimer, E. W. & Smucker, B. W. (2020). *IUCrData*, **5**(7), x200998. https://doi.org/10.1107/S2414314620009980

Howarth, A., Barbosa, T. J., Zeller, M. & Hillesheim, P. C. (2021). *IUCrData*, **6**(6), x210581. https://doi.org/10.1107/S2414314621005812

Hu, J., Nesterov, V. V. & Smucker, B. W. (2022*a*). *IUCrData*, **7**(2), x220101. https://doi.org/10.1107/S2414314622001018

Hu, Q., Wen, B. & Fan, C. (2021*a*). *IUCrData*, **6**(9), x210981. https://doi.org/10.1107/S2414314621009810

Hu, Q., Wen, B. & Fan, C. (2021*b*). *IUCrData*, **6**(9), x210988. https://doi.org/10.1107/S2414314621009883

Hu, Q., Wen, B. & Fan, C. (2022*b*). *IUCrData*, **7**(1), x220038. https://doi.org/10.1107/S2414314622000384

Huang, H. & Wu, Z. (2021). *IUCrData*, **6**(2), x201640. https://doi.org/10.1107/S2414314620016405

Ibragimov, A. (2020). *IUCrData*, **5**(7), x200843. https://doi.org/10.1107/S2414314620008433

Ichimaru, Y., Kato, K., Kurihara, M., Jin, W., Koike, T. & Kurosaki, H. (2022). *IUCrData*, **7**(8), x220854. https://doi.org/10.1107/S2414314622008549

Ichimaru, Y., Kato, K., Kurosaki, H., Fujioka, H., Sakai, M., Yamaguchi, Y., Wanchun, J., Sugiura, K., Imai, M. & Koike, T. (2021). *IUCrData*, **6**(4), x210397. https://doi.org/10.1107/S2414314621003977

Ishida, H. (2021). *IUCrData*, **6**(11), x211150. https://doi.org/10.1107/S2414314621011500

Ivlev, S. I. & Kraus, F. (2021). *IUCrData*, **6**(7), x210735. https://doi.org/10.1107/S2414314621007355

Jiang, H., Li, Y.-L., Zhou, J., Sun, H.-S., Zhang, Q.-Y., Shi, X.-H., Zhang, Z.-Y. & Ling, T. (2020). *IUCrData*, **5**(7), x200912. https://doi.org/10.1107/S2414314620009128

Johnston, D. H., King, C., Seitz, A. & Sethi, M. (2021). *IUCrData*, **6**(8), x210778. https://doi.org/10.1107/S2414314621007781

Kanagawa, M., Akagi, K. & Okuno, T. (2021). *IUCrData*, **6**(2), x210142. https://doi.org/10.1107/S2414314621001425

Kelley, S. P., Mossine, V. V. & Mawhinney, T. P. (2020). *IUCrData*, **5**(10), x201310. https://doi.org/10.1107/S2414314620013103

Khan, S. S., Howlader, M. B. H., Miyatake, R., Sheikh, M. C. & Zangrando, E. (2022). *IUCrData*, **7**(11), x221080. https://doi.org/10.1107/S241431462201080X

Khanum, G., Fatima, A., Sharma, P., Srivastava, S. K. & Butcher, R. J. (2021). *IUCrData*, **6**(4), x210351. https://doi.org/10.1107/S2414314621003515

Khelfa, S., Touil, M., Setifi, Z., Setifi, F., Al-Douh, M. H. & Glidewell, C. (2021). *IUCrData*, **6**(6), x210568. https://doi.org/10.1107/S241431462100568X

Kitada, A., Funasako, Y., Matsumoto, K., Hagiwara, R., Inokuchi, M., Fukami, K. & Murase, K. (2020). *IUCrData*, **5**(2), x200162. https://doi.org/10.1107/S2414314620001625

Kodama, S., Bunno, K., Nomoto, A. & Ogawa, A. (2021). *IUCrData*, **6**(10), x210992. https://doi.org/10.1107/S2414314621009925

Kodama, S., Kondo, S., Nomoto, A. & Ogawa, A. (2020). *IUCrData*, **5**(4), x200449. https://doi.org/10.1107/S2414314620004496

Koehne, S., Mirmelli, B., Mague, J. T. & Donahue, J. P. (2022). *IUCrData*, **7**(2), x220148. https://doi.org/10.1107/S2414314622001481

Koh, D. (2022). *IUCrData*, **7**(9), x220932. https://doi.org/10.1107/S2414314622009324

Kotha, S., Ansari, S. & Cheekatla, S. R. (2020*a*). *IUCrData*, **5**(10), x201380. https://doi.org/10.1107/S2414314620013802

Kotha, S., Ansari, S. & Gupta, N. K. (2021*a*). *IUCrData*, **6**(12), x211260. https://doi.org/10.1107/S2414314621012608

Kotha, S., Fatma, A. & Ansari, S. (2021*b*). *IUCrData*, **6**(2), x210167. https://doi.org/10.1107/S241431462100167X

Kotha, S., Gupta, N. K. & Ansari, S. (2020*b*). *IUCrData*, **5**(11), x201464. https://doi.org/10.1107/S2414314620014649

Kotha, S., Gupta, N. K. & Ansari, S. (2021*c*). *IUCrData*, **6**(4), x210322. https://doi.org/10.1107/S2414314621003229

Lambrecht, S., Villinger, A. & Jopp, S. (2022). *IUCrData*, **7**(3), x220265. https://doi.org/10.1107/S2414314622002656

Lesley, M. J. G., Ozhan, K., Sung, H. H.-Y. & Williams, I. D. (2020). *IUCrData*, **5**(3), x200417. https://doi.org/10.1107/S2414314620004174

Li, Y.-L., Zhou, J., Jiang, H., Sun, H.-S., Li, R.-Z., Liu, S.-L. & Zhang, X.-D. (2021). *IUCrData*, **6**(2), x210057. https://doi.org/10.1107/S2414314621000572

Liang, B.-B., Xiong, H.-G., Hong, W.-Y. & Yao, H.-G. (2020). *IUCrData*, **5**(11), x201433. https://doi.org/10.1107/S2414314620014339

Liebing, P. & Merzweiler, K. (2021). *IUCrData*, **6**(6), x210594. https://doi.org/10.1107/S2414314621005940

Lin, Z. & Li, J. (2020). *IUCrData*, **5**(7), x200877. https://doi.org/10.1107/S2414314620008779

Linkova, E. I., Grinev, V. S., Mayorova, O. A. & Yegorova, A. Y. (2020). *IUCrData*, **5**(7), x200919. https://doi.org/10.1107/S2414314620009190

Liu, L. & Han, Z.-B. (2022). *IUCrData*, **7**(1), x220046. https://doi.org/10.1107/S2414314622000463

Lough, A. J., Ho, A. & Tam, W. (2020*a*). *IUCrData*, **5**(2), x200265. https://doi.org/10.1107/S2414314620002655

Lough, A. J., Koh, S. & Tam, W. (2020*b*). *IUCrData*, **5**(3), x200315. https://doi.org/10.1107/S2414314620003156

Lough, A. J., Pounder, A. & Tam, W. (2020*c*). *IUCrData*, **5**(3), x200286. https://doi.org/10.1107/S2414314620002862

Lough, A. J., Pounder, A., Wicks, C. & Tam, W. (2020*d*). *IUCrData*, **5**(3), x200288. https://doi.org/10.1107/S2414314620002886

Lozinšek, M. (2021). *IUCrData*, **6**(11), x211215. https://doi.org/10.1107/S2414314621012153

Lutz, M. & Kroon-Batenburg, L. (2022). *IUCrData*, **7**(11), x221059. https://doi.org/10.1107/S2414314622010598

Martínez-de la Luz, I., López-Velázquez, D., Bernès, S. & Varela Caselis, J. L. (2022). *IUCrData*, **7**(9), x220919. https://doi.org/10.1107/S2414314622009191

Lynch, W. E., Whitlock, C. R. & Padgett, C. W. (2020). *IUCrData*, **5**(9), x201205. https://doi.org/10.1107/S2414314620012055

MacNeil, C. S., Ogweno, A. O., Ojwach, S. O. & Hayes, P. G. (2020). *IUCrData*, **5**(1), x200040. https://doi.org/10.1107/S2414314620000401

Majer, T., Schollmeyer, D., Koch, P. & Gross, H. (2020). *IUCrData*, **5**(12), x201578. https://doi.org/10.1107/S2414314620015783

Malan, F. P., Potgieter, K. & Meijboom, R. (2022*a*). *IUCrData*, **7**(11), x221045. https://doi.org/10.1107/S2414314622010458

Malan, F. P., Potgieter, K. & Meijboom, R. (2022*b*). *IUCrData*, **7**(12), x221148. https://doi.org/10.1107/S2414314622011488

Malin, A. V., Ivlev, S. I., Ostvald, R. V. & Kraus, F. (2020). *IUCrData*, **5**(1), x200114. https://doi.org/10.1107/S2414314620001145

Mallard, H. H., Kennedy, N. D., Rudman, N. A., Greenwood, A. M., Nicoleau, J., Angle, C. E., Torquato, N. A., Gau, M. R., Carroll, P. J. & Anstey, M. R. (2020). *IUCrData*, **5**(9), x201248. https://doi.org/10.1107/S2414314620012481

Mamadrakhimov, A., Mutalliyev, L., Abdullaev, S., Khodjaniyazov, K., Izotova, L. & Aisa, H. A. (2021). *IUCrData*, **6**(5), x210451. https://doi.org/10.1107/S241431462100451X

Mamallan, K., Gomathi, S., Soundararajan, K. & Sethuraman, V. (2020). *IUCrData*, **5**(1), x200011. https://doi.org/10.1107/S2414314620000115

Manickam, R., Jagadeesan, G., Karunakaran, J. & Srinivasan, G. (2020). *IUCrData*, **5**(2), x200212. https://doi.org/10.1107/S2414314620002126

Manjula, V., Venkateswaramoorthi, R., Dharmaraja, J. & Selvanayagam, S. (2020). *IUCrData*, **5**(4), x200526. https://doi.org/10.1107/S241431462000526X

Marolf, D. M., Brehm, K. L., Lynch, V. M. & Powell, G. L. (2020). *IUCrData*, **5**(7), x200935. https://doi.org/10.1107/S2414314620009359

Matsumoto, M., Nagayama, N., Hirose, R., Takeshita, K. & Ishii, T. (2021). *IUCrData*, **6**(12), x211325. https://doi.org/10.1107/S2414314621013250

Mayorova, O. A., Grinev, V. S. & Yegorova, A. Y. (2020). *IUCrData*, **5**(7), x200937. https://doi.org/10.1107/S2414314620009372

Meenatchi, C. S., Athimoolam, S., Suresh, J., Priya, R. V., Rubina, S. R. & Bhandari, S. R. (2022). *IUCrData*, **7**(3), x220283. https://doi.org/10.1107/S2414314622002838

Meenatchi, C. S., Athimoolam, S., Suresh, J., Rubina, S. R., Kumar, R. R. & Bhandari, S. R. (2021). *IUCrData*, **6**(11), x211195. https://doi.org/10.1107/S2414314621011950

Menia, D., Höfer, T., Wurst, K. & Bildstein, B. (2021*a*). *IUCrData*, **6**(5), x210460. https://doi.org/10.1107/S2414314621004600

Menia, D., Wurst, K. & Bildstein, B. (2021*b*). *IUCrData*, **6**(10), x211083. https://doi.org/10.1107/S241431462101083X

Miecznikowski, J. R., Jasinski, J. P., Flaherty, N. F., Mircovich, E. E., Smolinsky, A. N. & Bertolotti, N. R. (2020). *IUCrData*, **5**(9), x201182. https://doi.org/10.1107/S2414314620011827

Missioui, M., Guerrab, W., Alsubari, A., Mague, J. T. & Ramli, Y. (2022). *IUCrData*, **7**(7), x220621. https://doi.org/10.1107/S2414314622006216

Mizuhata, Y., Iwai, K. & Tokitoh, N. (2020). *IUCrData*, **5**(9), x201299. https://doi.org/10.1107/S2414314620012997

Mohamed-Ezzat, R. A., Kariuki, B. M. & Azzam, R. A. (2022). *IUCrData*, **7**(11), x221033. https://doi.org/10.1107/S2414314622010331

Morales-Collazo, O., Lynch, V. M. & Brennecke, J. F. (2020). *IUCrData*, **5**(5), x200681. https://doi.org/10.1107/S2414314620006811

Moris, S. & Galdámez, A. (2022). *IUCrData*, **7**(7), x220729. https://doi.org/10.1107/S2414314622007295

Morris, L. L., Alvarado, C. A., Goncalves, J. M., Singh, R. P., Lovely, C. J. & Yousufuddin, M. (2020). *IUCrData*, **5**(1), x200078. https://doi.org/10.1107/S2414314620000784

Mossine, V. V., Kelley, S. P. & Mawhinney, T. P. (2022). *IUCrData*, **7**(1), x220061. https://doi.org/10.1107/S241431462200061X

Moussa Slimane, N., Benarous, N., Bougueria, H. & Cherouana, A. (2022). *IUCrData*, **7**(2), x220112. https://doi.org/10.1107/S2414314622001122

Muller II, J. E., Osborn, L. R., Traver, J. R., Hillesheim, P. C., Zeller, M. & Mirjafari, A. (2020). *IUCrData*, **5**(2), x200170. https://doi.org/10.1107/S2414314620001704

Myers, S., Johnson, P. & Benedict, J. B. (2021). *IUCrData*, **6**(6), x210545. https://doi.org/10.1107/S2414314621005459

Naeem, M., Chadeayne, A. R., Golen, J. A. & Manke, D. R. (2022). *IUCrData*, **7**(4), x220364. https://doi.org/10.1107/S2414314622003649

Nagayama, N., Taniguchi, N., Matsumoto, M., Takeshita, K. & Ishii, T. (2020). *IUCrData*, **5**(12), x201625. https://doi.org/10.1107/S2414314620016259

Narayan, E., Fu, L., Gribble, G. W., Kaur, M. & Jasinski, J. P. (2020). *IUCrData*, **5**(3), x200382. https://doi.org/10.1107/S241431462000382X

Narvekar, K. U. & Srinivasan, B. R. (2020*a*). *IUCrData*, **5**(2), x200230. https://doi.org/10.1107/S2414314620002308

Narvekar, K. U. & Srinivasan, B. R. (2020*b*). *IUCrData*, **5**(1), x200100. https://doi.org/10.1107/S2414314620001005

Nazarenko, A. Y. (2020). *IUCrData*, **5**(12), x201646. https://doi.org/10.1107/S2414314620016466

Neal, H. C., Nesterov, V. V. & Smucker, B. W. (2022). *IUCrData*, **7**(5), x220525. https://doi.org/10.1107/S2414314622005259

Newell, B. D., McMillen, C. D. & Lee, J. P. (2022). *IUCrData*, **7**(8), x220804. https://doi.org/10.1107/S2414314622008045

Newman, E. B., Astashkin, A. V., Albert, D. R. & Rajaseelan, E. (2021). *IUCrData*, **6**(8), x210836. https://doi.org/10.1107/S2414314621008361

Noland, W. E., Herzig, R. J., Fox, R. J. & Tritch, K. J. (2021). *IUCrData*, **6**(4), x210391. https://doi.org/10.1107/S2414314621003916

Obidova, N., Ashurov, J., Izotova, L. & Ibragimov, B. (2022). *IUCrData*, **7**(4), x220441. https://doi.org/10.1107/S2414314622004412

Okuno, T. & Doi, I. (2022). *IUCrData*, **7**(9), x220942. https://doi.org/10.1107/S2414314622009427

Olszewski, A. & Wzgarda-Raj, K. (2021). *IUCrData*, **6**(10), x211102. https://doi.org/10.1107/S2414314621011020

Outahar, F., Moumou, M., Rakib, E. M., Hannioui, A., Saadi, M. & El Ammari, L. (2020). *IUCrData*, **5**(7), x200945. https://doi.org/10.1107/S2414314620009451

Ovalle, M. A., Romero, J. A. & Aguirre, G. (2021). *IUCrData*, **6**(1), x201663. https://doi.org/10.1107/S2414314620016636

Pacifico, J. & Stoeckli-Evans, H. (2021). *IUCrData*, **6**(12), x211295. https://doi.org/10.1107/S2414314621012955

Pacifico, J. & Stoeckli-Evans, H. (2022). *IUCrData*, **7**(2), x220077. https://doi.org/10.1107/S2414314622000773

Padgett, C. W., Sheriff, K. & Lynch, W. E. (2020). *IUCrData*, **5**(10), x201335. https://doi.org/10.1107/S2414314620013358

Papa, V., Spannenberg, A., Beller, M. & Junge, K. (2020). *IUCrData*, **5**(12), x201570. https://doi.org/10.1107/S2414314620015709

Patel, D. G., Cox, J. M., Bender, B. M. & Benedict, J. B. (2021). *IUCrData*, **6**(10), x211016. https://doi.org/10.1107/S2414314621010166

Peppel, T. & Köckerling, M. (2021*a*). *IUCrData*, **6**(8), x210818. https://doi.org/10.1107/S241431462100818X

Peppel, T. & Köckerling, M. (2021*b*). *IUCrData*, **6**(6), x210562. https://doi.org/10.1107/S2414314621005629

Peppel, T., Wulf, C. & Spannenberg, A. (2020). *IUCrData*, **5**(6), x200768. https://doi.org/10.1107/S2414314620007683

Pérez-Benítez, A., Ariza-Ramírez, J. L., Fortis-Valera, M., Arroyo-Carmona, R. E., Martínez de la Luz, M. I., Ramírez-Contreras, D. & Bernès, S. (2022). *IUCrData*, **7**(6), x220627. https://doi.org/10.1107/S2414314622006277

Pérez-Benítez, A. & Bernès, S. (2020). *IUCrData*, **5**(4), x200488. https://doi.org/10.1107/S2414314620004885

Pham, D. N. K., Chadeayne, A. R., Golen, J. A. & Manke, D. R. (2021). *IUCrData*, **6**(2), x210123. https://doi.org/10.1107/S2414314621001231

Pineda, L. W., Ferllini, N. & Cabezas, J. A. (2022). *IUCrData*, **7**(4), x220401. https://doi.org/10.1107/S2414314622004011

Polito-Lucas, J. A., Núñez-Ávila, J. A., Bernès, S. & Pérez-Benítez, A. (2021). *IUCrData*, **6**(6), x210634. https://doi.org/10.1107/S2414314621006349

Potgieter, K., Malan, F. P., Alimi, O. A. & Meijboom, R. (2022*a*). *IUCrData*, **7**(8), x220771. https://doi.org/10.1107/S2414314622007714

Potgieter, K., Malan, F. P., Alimi, O. A. & Meijboom, R. (2022*b*). *IUCrData*, **7**(12), x221147. https://doi.org/10.1107/S2414314622011476

Potgieter, K., Malan, F. P. & Meijboom, R. (2022*c*). *IUCrData*, **7**(8), x220772. https://doi.org/10.1107/S2414314622007726

Powell, G. L. & Rix, B. A. (2020). *IUCrData*, **5**(2), x200121. https://doi.org/10.1107/S2414314620001212

Priyanka, P., Jayanna, B. K., Kiran Kumar, H., Yathirajan, H. S., Divakara, T. R., Foro, S. & Butcher, R. J. (2022). *IUCrData*, **7**(9), x220904. https://doi.org/10.1107/S241431462200904X

Purdy, S. K., Spasyuk, D., Chitanda, J. M. & Reaney, M. J. T. (2020). *IUCrData*, **5**(3), x200318. https://doi.org/10.1107/S2414314620003181

Ravisankar, V., Ramesh, V., Krishnamohan, M., Gunasekaran, B. & Girisun, T. C. S. (2021). *IUCrData*, **6**(1), x210024. https://doi.org/10.1107/S2414314621000249

Reed, C. R., Garner, R. N. & Brennessel, W. W. (2021). *IUCrData*, **6**(3). https://doi.org/10.1107/S241431462100287X

Ren, J.-Y., Huang, R., Yin, Z. & Cao, L.-H. (2022). *IUCrData*, **7**(8), x220775. https://doi.org/10.1107/S2414314622007751

Ren, W. & Li, J. (2022). *IUCrData*, **7**(9), x220869. https://doi.org/10.1107/S2414314622008690

Rincke, C., Schmidt, H. & Voigt, W. (2020). *IUCrData*, **5**(2), x200165. https://doi.org/10.1107/S2414314620001650

Rood, J. A., Subedi, C. B., Risell, J. P., Astashkin, A. V. & Rajaseelan, E. (2021). *IUCrData*, **6**(6), x210597. https://doi.org/10.1107/S2414314621005976

Rushlow, J., Astashkin, A. V., Albert, D. R. & Rajaseelan, E. (2021). *IUCrData*, **6**(8), x210811. https://doi.org/10.1107/S2414314621008117

Rushlow, J., Astashkin, A. V., Albert, D. R. & Rajaseelan, E. (2022). *IUCrData*, **7**(7), x220685. https://doi.org/10.1107/S241431462200685X

Saadallah, Y., Setifi, Z., Geiger, D. K., Al-Douh, M. H., Satour, A. & Setifi, F. (2022). *IUCrData*, **7**(12), x221180. https://doi.org/10.1107/S2414314622011804

Saha, A., Padgett, C. W., LeMagueres, P., Moncur, K. & Onajobi, G. (2021). *IUCrData*, **6**(1), x201643. https://doi.org/10.1107/S2414314620016430

Saini, A., Dhanwant, K. & Thirumoorthi, R. (2022). *IUCrData*, **7**(10), x221023. https://doi.org/10.1107/S2414314622010239

Salerno, E. V., Kadel, L. R. & Eichhorn, D. M. (2021). *IUCrData*, **6**(7), x210690. https://doi.org/10.1107/S2414314621006908

Sammeta, V. R., Rasapalli, S., Chadeayne, A. R., Golen, J. A. & Manke, D. R. (2020). *IUCrData*, **5**(11), x201546. https://doi.org/10.1107/S2414314620015461

Samoľová, E., Dehno Khalaji, A. & Eigner, V. (2021*a*). *IUCrData*, **6**(4), x210335. https://doi.org/10.1107/S2414314621003357

Samoľová, E., Dehno Khalaji, A. & Eigner, V. (2021*b*). *IUCrData*, **6**(4), x210356. https://doi.org/10.1107/S2414314621003564

Samolová, E. & Fábry, J. (2020). *IUCrData*, **5**(10), x201311. https://doi.org/10.1107/S2414314620013115

Sánchez-García, J. J., Flores-Alamo, M., Nuñez-Gordillo, A. & Klimova, E. I. (2022). *IUCrData*, **7**(10), x221011. https://doi.org/10.1107/S2414314622010112

Sathya, U., Nirmal Ram, J. S., Gomathi, S., Jegan Jennifer, S. & Abdul Razak, I. (2021*a*). *IUCrData*, **6**(5), x210522. https://doi.org/10.1107/S2414314621005228

Sathya, U., Nirmal Ram, J. S., Gomathi, S., Ramu, S., Jegan Jennifer, S. & Ibrahim, A. R. (2021*b*). *IUCrData*, **6**(4), x210379. https://doi.org/10.1107/S2414314621003795

Schmidt, A.-C., Iovkova, L. & Hiersemann, M. (2021). *IUCrData*, **6**(9), x210951. https://doi.org/10.1107/S2414314621009512

Schmitt, V., Holzmann, G., Schollmeyer, D. & Detert, H. (2021). *IUCrData*, **6**(4), x210443. https://doi.org/10.1107/S2414314621004430

Schödel, F., Lerner, H.-W. & Bolte, M. (2020). *IUCrData*, **5**(6), x200863. https://doi.org/10.1107/S2414314620008639

Schollmeyer, D. & Detert, H. (2022). *IUCrData*, **7**(2), x220169. https://doi.org/10.1107/S2414314622001699

Schollmeyer, D., Heidrich, M. & Detert, H. (2020). *IUCrData*, **5**(9), x201302. https://doi.org/10.1107/S2414314620013024

Schollmeyer, D., Sadovski, O. & Detert, H. (2021). *IUCrData*, **6**(6), x210654. https://doi.org/10.1107/S2414314621006544

Schröder, F. & Köckerling, M. (2021*a*). *IUCrData*, **6**(3), x210304. https://doi.org/10.1107/S2414314621003047
Schröder, F. & Köckerling, M. (2021*b*). *IUCrData*, **6**(7), x210696. https://doi.org/10.1107/S2414314621006969
Schröder, F. & Köckerling, M. (2022). *IUCrData*, **7**(6), x220618. https://doi.org/10.1107/S2414314622006186
Schuster, S. A., Nesterov, V. V. & Smucker, B. W. (2022). *IUCrData*, **7**(5), x220526. https://doi.org/10.1107/S2414314622005260
Setifi, Z., Setifi, F., Dege, N., Al-Douh, M. H. & Glidewell, C. (2020). *IUCrData*, **5**(9), x201278. https://doi.org/10.1107/S241431462001278X
Sha, F. & Johnson, A. R. (2020). *IUCrData*, **5**(12), x201580. https://doi.org/10.1107/S2414314620015801
Shahri, N. N. M., Omar Ali, N. H. S., Sheikh Abdul Hamid, M. H., Mirza, A. H., Usman, A., Hoq, M. R. & Karim, M. R. (2020). *IUCrData*, **5**(2), x200134. https://doi.org/10.1107/S2414314620001340
Shank, N., Stadler, A. L., Barrett, S. P. & Padgett, C. W. (2022). *IUCrData*, **7**(8), x220797. https://doi.org/10.1107/S2414314622007970
Shevlin, M. R., Stumbo, E. E., McMillen, C. D. & Pienkos, J. A. (2022). *IUCrData*, **7**(8), x220830. https://doi.org/10.1107/S2414314622008306
Shimazaki, R. & Sadakiyo, M. (2022*a*). *IUCrData*, **7**(9), x220884. https://doi.org/10.1107/S2414314622008847
Shimazaki, R. & Sadakiyo, M. (2022*b*). *IUCrData*, **7**(10), x220951. https://doi.org/10.1107/S2414314622009518
Shirmila, D. A., Jonathan, D. R., Priya, M. K., Hemalatha, J. & Usha, G. (2021). *IUCrData*, **6**(3), x210309. https://doi.org/10.1107/S2414314621003096
Show, V. L., Fok, E. Y. & Johnson, A. R. (2020). *IUCrData*, **5**(12), x201576. https://doi.org/10.1107/S241431462001576X
Shraddha, K. N. & Begum, N. S. (2020). *IUCrData*, **5**(7), x200870. https://doi.org/10.1107/S2414314620008706
Shraddha, K. N., Devika, S. & Begum, N. S. (2020). *IUCrData*, **5**(1), x191690. https://doi.org/10.1107/S2414314619016900
Shripanavar, C. S. & Butcher, R. J. (2020). *IUCrData*, **5**(8), x201060. https://doi.org/10.1107/S2414314620010603
Siddiqui, M. J., Nesterov, V. V., Steidle, M. T. & Smucker, B. W. (2020). *IUCrData*, **5**(7), x200980. https://doi.org/10.1107/S2414314620009803
Siegel, D. J., Howarth, A. N., Traver, J. R., Hillesheim, P. C., Zeller, M. & Mirjafari, A. (2020). *IUCrData*, **5**(2), x200171. https://doi.org/10.1107/S2414314620001716
Sivapriya, S., Priyanka, S., Gopalakrishnan, M., Manikandan, H. & Selvanayagam, S. (2021). *IUCrData*, **6**(5), x210500. https://doi.org/10.1107/S2414314621005009
Smith, T. J., Koser, G., Chen, Y., Zeller, M., Iacino, R. & Selzer, N. (2022). *IUCrData*, **7**(5), x220522. https://doi.org/10.1107/S2414314622005223
Song, J., Jiang, X., Wang, Z., Pei, J. & Li, H. (2022). *IUCrData*, **7**(3), x220342. https://doi.org/10.1107/S241431462200342X
Soundararajan, K., Sethuraman, V. & Thanigaimani, K. (2020). *IUCrData*, **5**(2), x200143. https://doi.org/10.1107/S2414314620001431
Srinivasan, B. R., Bhargao, P. H. & Sudhadevi, P. K. (2020*a*). *IUCrData*, **5**(4), x200448. https://doi.org/10.1107/S2414314620004484
Srinivasan, B. R., Harmalkar, S. S., D'Souza, L. R. & Dhuri, S. N. (2020*b*). *IUCrData*, **5**(6), x200796. https://doi.org/10.1107/S2414314620007968
Srinivasan, B. R., Parsekar, N. U. & Narvekar, K. U. (2020*c*). *IUCrData*, **5**(11), x201498. https://doi.org/10.1107/S2414314620014984
Stammler, H.-G. & Imran, M. (2020). *IUCrData*, **5**(1), x200067. https://doi.org/10.1107/S241431462000067X
Su, W., Fu, T. & Xu, Z. (2021). *IUCrData*, **6**(7), x210693. https://doi.org/10.1107/S2414314621006933
Sung, J. (2020). *IUCrData*, **5**(9), x201209. https://doi.org/10.1107/S2414314620012092
Sung, J. (2021). *IUCrData*, **6**(9), x210950. https://doi.org/10.1107/S2414314621009500
Sung, J. (2022). *IUCrData*, **7**(9), x220885. https://doi.org/10.1107/S2414314622008859
Surya Prakash Rao, H., M, P. & Muthukumaran, J. (2022). *IUCrData*, **7**(2), x220199. https://doi.org/10.1107/S2414314622001997
Taeufer, T., Spannenberg, A. & Pospech, J. (2021). *IUCrData*, **6**(10), x211026. https://doi.org/10.1107/S2414314621010269
Thiruvalluvar, A. A., Kusanur, R. & Sridharan, M. (2021). *IUCrData*, **6**(7), x210694. https://doi.org/10.1107/S2414314621006945
Tiouabi, M., Tabacchi, R. & Stoeckli-Evans, H. (2020). *IUCrData*, **5**(10), x201391. https://doi.org/10.1107/S2414314620013917
Tojiboev, A., Nasrullaev, A., Turgunov, K., Elmuradov, B. & Tashkhodjaev, B. (2020). *IUCrData*, **5**(3). https://doi.org/10.1107/S2414314620003569
Uppu, S. N., Agu, O. A., Deere, C. J. & Fronczek, F. R. (2020). *IUCrData*, **5**(8), x201121. https://doi.org/10.1107/S2414314620011219
Uzorka, B. & LaDuca, R. L. (2020). *IUCrData*, **5**(5), x200589. https://doi.org/10.1107/S2414314620005891
Vinaya, Basavaraju, Y. B., Nagma Banu, H. A., Kalluraya, B., Yathirajan, H. S., Balerao, R. & Butcher, R. J. (2022). *IUCrData*, **7**(10), x220957. https://doi.org/10.1107/S2414314622009579
Vinotha, G., Sundar, T. V. & Sharmila, N. (2021). *IUCrData*, **6**(2), x210210. https://doi.org/10.1107/S2414314621002108
Wang, S., Han, H. & Han, Y. (2020). *IUCrData*, **5**(9), x201240. https://doi.org/10.1107/S2414314620012407
Wang, S., Liu, G. & Wu, Z. (2021). *IUCrData*, **6**(6), x210396. https://doi.org/10.1107/S2414314621003965
Wang, Z. (2020). *IUCrData*, **5**(1), x191731. https://doi.org/10.1107/S2414314619017310
Weil, M., Kremsmayr, T. & Mihovilovic, M. D. (2020). *IUCrData*, **5**(2), x200224. https://doi.org/10.1107/S2414314620002242
Welton, C. E., Nesterov, V. N. & Smucker, B. W. (2022). *IUCrData*, **7**(3), x220248. https://doi.org/10.1107/S2414314622002486
Whalen, A. C., Hernandez Brito, C., Choi, K. H., Warner, E. J. T., Thole, D. A., Gau, M. R., Carroll, P. J. & Anstey, M. R. (2020). *IUCrData*, **5**(9), x201276. https://doi.org/10.1107/S2414314620012766
Wu, M.-F., Chen, L.-Y. & Li, Y. (2020). *IUCrData*, **5**(7), x201028. https://doi.org/10.1107/S2414314620010287
Xu, D., Gao, J. & Long, S. (2020). *IUCrData*, **5**(6), x200801. https://doi.org/10.1107/S2414314620008019
Yaffa, L., Pouye, S. F., Ndoye, D., Diallo, W., Diop, M., Sidibe, M. & Diop, C. A. K. (2021). *IUCrData*, **6**(9), x210982. https://doi.org/10.1107/S2414314621009822
Yan, D. (2021). *IUCrData*, **6**(4), x210428. https://doi.org/10.1107/S2414314621004284
Yang, X. & Long, S. (2021). *IUCrData*, **6**(5), x210539. https://doi.org/10.1107/S2414314621005393
Yoo, M. & Koh, D. (2020*a*). *IUCrData*, **5**(1), x200034. https://doi.org/10.1107/S2414314620000346
Yoo, M. & Koh, D. (2020*b*). *IUCrData*, **5**(1), x200071. https://doi.org/10.1107/S2414314620000711
Yoo, M. & Koh, D. (2020*c*). *IUCrData*, **5**(6), x200792. https://doi.org/10.1107/S2414314620007920
Yoo, M. & Koh, D. (2021*a*). *IUCrData*, **6**(1), x210096. https://doi.org/10.1107/S2414314621000961
Yoo, M. & Koh, D. (2021*b*). *IUCrData*, **6**(6), x210590. https://doi.org/10.1107/S2414314621005903

Yu, M., Wang, L., Wang, L. & Wu, Z. (2020). *IUCrData*, **5**(5), x200603. https://doi.org/10.1107/S2414314620006033

Yuan, Y. & Li, J. (2022). *IUCrData*, **7**(3), x220241. https://doi.org/10.1107/S2414314622002413

Zaoui, Y., Assila, H., Mague, J. T., Alsubari, A., Taoufik, J., Ramli, Y. & Ansar, M. (2022). *IUCrData*, **7**(6), x220582. https://doi.org/10.1107/S241431462200582X

Zhang, W., Zhang, B. & Sun, Q. (2021). *IUCrData*, **6**(7), x210672. https://doi.org/10.1107/S2414314621006726

Zhou, Y., Fan, C., Wen, B. & Zhang, L. (2022). *IUCrData*, **7**(7), x220694. https://doi.org/10.1107/S2414314622006940

Zometa Paniagua, D. F., Powell, G. L., Powell, C. B. & Reinheimer, E. W. (2020). *IUCrData*, **5**(9), x201204. https://doi.org/10.1107/S2414314620012043
